# In Silico Models for Predicting Adsorption of Organic Pollutants on Atmospheric Nanoplastics by Combining Grand Canonical Monte Carlo/Density Functional Theory and Quantitative Structure Activity Relationship Approach

**DOI:** 10.3390/nano16030178

**Published:** 2026-01-28

**Authors:** Ya Wang, Honghong Yi, Chao Li, Xiaolong Tang, Peng Zhao, Zhongfang Chen

**Affiliations:** 1 School of Energy and Environmental Engineering, University of Science and Technology Beijing, Beijing 100083, China; wyanne@ustb.edu.cn (Y.W.);; 2 Engineering Laboratory for Water Pollution Control and Resources Recovery, School of Environment, Northeast Normal University, Changchun 130117, China; 3 State Environmental Protection Key Laboratory of Wetland Ecology and Vegetation Restoration, School of Environment, Northeast Normal University, Changchun 130117, China; 4 Department of Chemistry, University of Puerto Rico, San Juan, PR 00931, USA

**Keywords:** nanoplastics, adsorption equilibrium constant, adsorption capacity, grand canonical Monte Carlo (GCMC) simulation, density functional theory (DFT) calculations, predictive model

## Abstract

Estimating the adsorption data and understanding the adsorption behavior and mechanism of organic pollutants on nanoplastics are crucial for assessing their ecological risks. Herein, in silico techniques, i.e., grand canonical Monte Carlo simulations, density functional theory computations, and quantitative structure activity relationship (QSAR) modeling, were integrated to examine the adsorption of 39 representative aliphatic and aromatic compounds and nine emerging pollutants (brominated flame retardants and phosphorus flame retardants) onto 12 different nanoplastics under atmospheric conditions. Three QSAR models were constructed to predict the adsorption equilibrium constant (log*K*) for polyethylene, polyoxymethylene, and polyvinyl alcohol nanoplastics individually, along with 12 QSAR models for separately estimating adsorption capacities (*C*_m_) on different nanoplastics. Furthermore, a novel multi-dimensional prediction model was developed, enabling simultaneous, high-throughput prediction of adsorption capacities across multiple nanoplastics and pollutants with a single input. These results revealed that van der Waals and electrostatic interactions serve as the primary driving forces for the adsorption. The novel multi-dimensional prediction model facilitates rapid and comprehensive assessment of pollutant–nanoplastic interactions with one-click, and paves the way for improved risk evaluations and advancing predictive environmental research.

## 1. Introduction

The synthesis of plastics dates back to the early 20th century, and their production and use have grown exponentially since the 1950s [1]. It is estimated that the global plastic production exceeded 400 million metric tons (Mt) in 2022, and will more than double by 2050 compare d with 2020 levels [2,3]. Alarmingly, only ~9% of plastics are recycled and ~12% are incinerated, leaving ~79% deposited of in landfills or released into the environment [1,4]. Through natural weathering in the environment, larger plastic debris progressively fragments into microplastics (1 μm to 5 mm) and ultimately into nanoplastics (NPs, <1 μm) [5], which have been widely detected across various environmental media, including water, air, soil, and biosphere [6,7,8]. Owing to their hydrophobic nature and high specific surface area, NPs tend to adsorb organic pollutants, and can act as vectors that facilitate pollutant transport in the environment [9,10,11], thereby influencing pollutant fate and potentially generating ecological risks. Therefore, it is critical to understand the adsorption mechanisms of organic pollutants on NPs, and evaluate their environmental behavior and associated ecological risks.

Despite its importance, research on the adsorption of organic pollutants onto NPs is still in its infancy, with limited experimental data available. Existing experimental studies have investigated the adsorption of various organic compounds onto diverse microplastics/NPs in an aqueous environment, uncovering heterogeneous and complex adsorption mechanisms. A single microplastic or NP can exhibit varying adsorption capabilities for different organic pollutants. For instance, the adsorption capacities of polyethylene microplastic increase sequentially for carbamazepine, 17α-ethinyl estradiol, triclosan, and 4-methylbenzylidene camphor [12]. Similarly, different microplastics/NPs show different adsorption capabilities for the same pollutant. For example, polystyrene nanoplastic exhibits stronger adsorption for polychlorinated biphenyl compared to polyethylene microplastic [13]. Additionally, surface functionalization of plastics, such as functional groups or attached biofilms, can affect the adsorption behavior [14,15]. Moreover, environmental factors, such as temperature [16], pH [17], and dissolved organic matter [18] in the water environment also play critical roles.

Typically, multiple interactions including π-π interactions, hydrophobic forces, dispersion, electrostatic interactions, and hydrogen bonding coexist during the adsorption of organic pollutants onto microplastics/NPs, and these interactions depend on the structures and properties of both microplastics/NPs and organic pollutants [19,20,21]. Significant insights have been gained from studies in aqueous environments [22,23], while research on how the structures and properties of NPs and organic pollutants influence these interactions in the atmosphere remains limited. Therefore, it is crucial to elucidate the adsorption mechanism of diverse organic pollutants on NPs in the atmospheric conditions.

Compared with experimental methods, computational approaches offer significant advantages for elucidating adsorption mechanisms at the atomic level. These techniques provide detailed insights into the interactions between NPs and pollutants, enabling systematic interrogation of adsorption processes that are often challenging to isolate or directly observe experimentally. Therefore, computational investigations of pollutant adsorption on NPs have gained increasing popularity and represent a growing frontier in environmental research [24].

Various computational methods, such as grand canonical Monte Carlo (GCMC), quantum chemistry, and molecular dynamics, have been applied to explore the adsorption of organic pollutants on NPs, including polyethylene, polypropylene, and polyvinyl chloride [25,26]. Each method offers distinct characteristics and advantages. GCMC simulations [27,28] can be employed to obtain the adsorption isotherms and predict adsorption capacities based on the probabilistic statistical principles; however, they cannot provide direct insights into the adsorption dynamics. In contrast, molecular dynamics simulations enable the modeling of dynamic adsorption processes. However, both GCMC and molecular dynamics cannot provide accurate electronic properties, while quantum chemical approaches, particularly density-functional theory (DFT) [29,30], can provide us accurate adsorption energies along detailed electronic properties. Nevertheless, DFT calculations entail higher computational costs than either GCMC or molecular dynamics simulations.

Given the vast number of organic pollutants and diverse NPs in the environment, it is not practical, if not impossible, to simulate the adsorption process individually for each pollutant–NP pair. Hence, it is urgent to leverage simulation methods and develop predictive models to efficiently and accurately estimate adsorption data, thereby deepening our understanding of pollutant–NP interactions in the atmosphere. Quantitative structure activity relationship (QSAR) techniques have been successfully utilized to establish predictive models for adsorption of organic pollutants on microplastics in aquatic environments [31,32,33,34,35]. However, these models are largely restricted to aqueous systems, and equivalent models tailored to gas-phase atmospheric conditions remain scarce. Therefore, there is an urgent need to develop novel predictive models to estimate the adsorption of organic pollutants towards NPs in the atmosphere.

In this work, 39 representative aliphatic and aromatic compounds, along with nine emerging pollutants, including brominated flame retardants and phosphorus flame retardants, were selected as adsorbates, while 12 different nanoplastics were chosen as adsorbents. Using GCMC simulations, we estimated the adsorption capacities (*C*_m_) of these 48 organic pollutants on the 12 NPs under atmospheric conditions. Subsequently, DFT calculations were carried out to obtain the logarithm of the adsorption equilibrium coefficients (log*K*) of organic pollutants on three NPs, namely, polyethylene (PE), polyoxymethylene (POM), and polyvinyl alcohol (PVA), since they exhibited the highest adsorption capacities among these 12 NPs. We also developed three QSAR models for predicting the log*K* values of organic pollutants on PE, POM and PVA individually, as well as 12 QSAR models for predicting the *C*_m_ values for each NP. Based on these 12 QSAR models, we established a novel multi-dimensional QSAR model incorporating parameters describing the structures of both organic pollutants and NPs, enabling simultaneous prediction of adsorption capacities across diverse organic pollutants and NPs in the atmosphere. These QSAR models not only advance mechanistic understanding of adsorption on NPs in atmospheric environments, but also provide efficient tools for generating adsorption data that are critical for assessing the ecological risks associated with organic pollutants and NPs.

## 2. Computational Details

### 2.1. Adsorbent and Adsorbate Models

**Figure 1:**
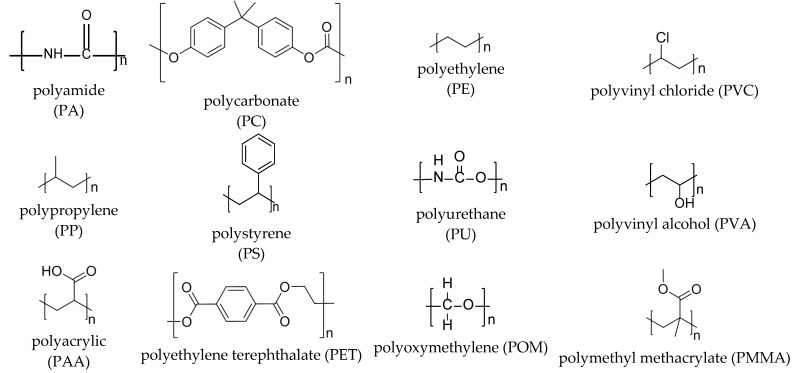
Twelve model nanoplastics.

Twelve different nanoplastics (Figure 1) were constructed as adsorbent models in a periodic simulation cell of 25 Å × 25 Å × 50 Å. Each nanoplastic structure was designed with the corresponding cell length of 50 Å along the Z-direction so as to provide sufficient gas-phase space for adsorption sampling and to reduce interactions between periodic images along the surface-normal direction. As exhibited in Figure 1, among these twelve nanoplastics, PC, PS and PET contain phenyl rings, while the remaining nine nanoplastics do not. All nanoplastic structures were fully geometry-relaxed prior to GCMC simulations; the non-linear conformation of PVA originates from the stabilized hydrogen-bonding network of hydroxyl groups, rather than from particle deformation or clustering”.

The adsorbates comprised 48 different organic compounds, including 39 representative aliphatic and aromatic compounds with different functional groups, i.e., -OH, -NH_2_, -NO_2_, -CH_3_, -CN, -CHO, -CH_2_OH, -CH_2_CH_3_, -C(O)CH_3_, -CH_2_CH_2_OH, -OC(O)CH_3_, -C(O)OCH_3_, -CH_2_CH_2_CH_3_, -C(O)OCH_2_CH_3_, -F and -C_6_H_5_, and nine emerging pollutants (including brominated flame retardants and phosphorus flame retardants), as listed in Table S1 of the Supplementary Material.

### 2.2. Grand Canonical Monte Carlo Simulation

All the GCMC simulations were conducted under the grand canonical ensemble using the Sorption program [36]. The Metropolis algorithm [37] was used to ensure sufficient sampling. The COMPASS II forcefield [38], which extends the coverage for the COMPASS force field [39,40,41] to polymer and drug-like molecules, was utilized for all the adsorption isotherm simulations. Fugacity values ranged from 101.325 kPa to 1013.25 kPa, and the temperature was 298 K. The upper fugacity values were used to construct complete adsorption isotherms and identify saturation behavior, rather than to represent typical ambient atmospheric pressure. All pollutants and nanoplastics were evaluated under the same gas-phase conditions (298 K; no solvent; pH not applicable), and the 101.325 kPa fugacity is used for environmental discussion unless mentioned otherwise. The electrostatic interactions were calculated using the Ewald & Group based method with a cutoff distance of 12.5 Å [42], while the van der Waals interactions were obtained using the atom-based method with the same cutoff distance of 12.5 Å and a spline width of 1 Å [38,43,44,45,46]. All Lennard–Jones parameters and fixed partial charges were assigned from the COMPASS force field without reparameterization.

### 2.3. Density Functional Theory Computation

Among these 12 nanoplastics, those with adsorption capacities (*C*_m_) in the top 30%, as determined by GCMC simulations, were selected for further estimating their adsorption equilibrium coefficient (*K*) values using DFT calculations. All DFT calculations were carried out with the DMol^3^ program [47,48]. The default temperature was set to 0 K, and no solvent effects was considered for these DFT computations. The Perdew–Burke–Ernzerhof (PBE) functional within the generalized gradient approximation (GGA) was employed [49], along with the double-numerical basis with polarization functions (DNP) [50,51], which is comparable to Gaussian 6-31G(d, p) [52]. Additionally, to account for long-range interactions, the PBE + D2 method with the Grimme van der Waals (vdW) correction [53] was applied.

### 2.4. Adsorption Data and Molecular Structure Descriptors

To examine the adsorption behavior, we calculated both *C*_m_ and *K* values. *C*_m_ represents the mass of organic compounds adsorbed per unit mass of the adsorbent, and can be obtained from the GCMC simulations. In contrast, the *K* values, representing the adsorption equilibrium coefficient, can be calculated as follows [54]:

(1) $$K=e^{-\frac{\Delta G}{RT}}$$

where *T* is the temperature, set at 298.15 K; *R* is the universal gas constant (8.314 J·mol^−1^·K^−1^). Δ*G* represents the change in Gibbs free energy, and can be estimated with the following equation [55]:

Δ*G* = Δ*E* + Δ*ZPE* − *T*Δ*S* (2)

where Δ*E* is the changes in total energy, Δ*ZPE* is the changes in zero-point energy, and Δ*S* is the changes in entropy. Δ*E*, Δ*ZPE*, and Δ*S* can be obtained from the DFT computations.

The molecular structures of the 12 nanoplastic monomers and 48 organic pollutants were fully optimized using DFT. Based on these 48 optimized pollutant structures, 2325 molecular structural descriptors and fingerprints were generated using PaDEL software (http://padel.nus.edu.sg/software/padeldescriptor, accessed on 17 May 2024) [56]. Descriptors and fingerprints with a zero value were removed, resulting in 643 relevant parameters for developing QSAR models. Similarly, we calculated the descriptors and fingerprints for the 12 nanoplastics using their optimized structures. These descriptors and fingerprints encode the structural characteristics of these 12 nanoplastics and were therefore used in establishing the QSAR models.

### 2.5. Establishment and Evaluation for QSAR Models

For the development of QSAR models to predict log*K* values, these 48 organic compounds were randomly divided into a training set of 40 compounds and a validation set of eight compounds. In contrast, for the QSAR models predicting *C*_m_ values, all the 48 organic compounds were used to construct the models without external validation. Multiple linear regression (MLR) analysis was conducted to construct QSAR models. We further evaluated the goodness-of-fit, robustness, and predictive capability of the established QSAR models using standard statistic metrics, including the adjusted determination coefficient (*R*^2^_adj_), root mean square error for the training set (*RMSE*_t_), and, for the validation set, *RMSE*_v_, the external determination coefficient (*R*^2^_ext_), and the external explained variance (*Q*^2^_ext_).

Additionally, we used Williams plots, which are based on standardized residuals (δ^*^) and leverage values (*h*_i_) [57], to characterize the application domain (AD) of the developed prediction models.

## 3. Results and Discussion

### 3.1. C_m_ and logK Values for Organic Compounds on Nanoplastics

**Figure 2:**
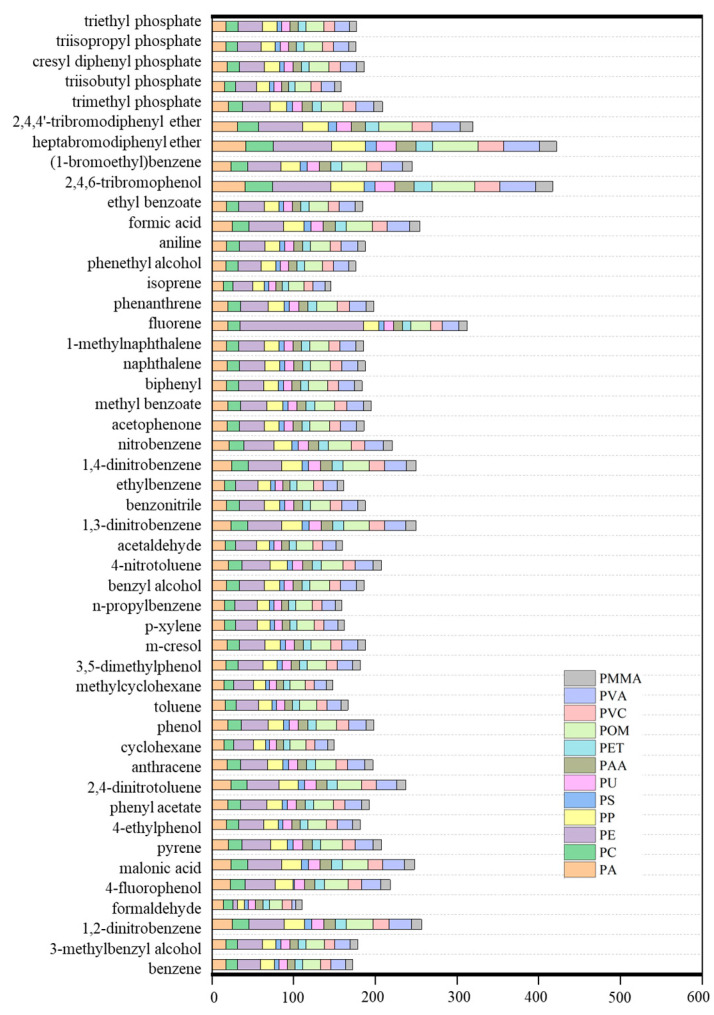
*C*_m_ values (g/g) for 48 organic compounds on 12 nanoplastics from GCMC simulations.

**Figure 3:**
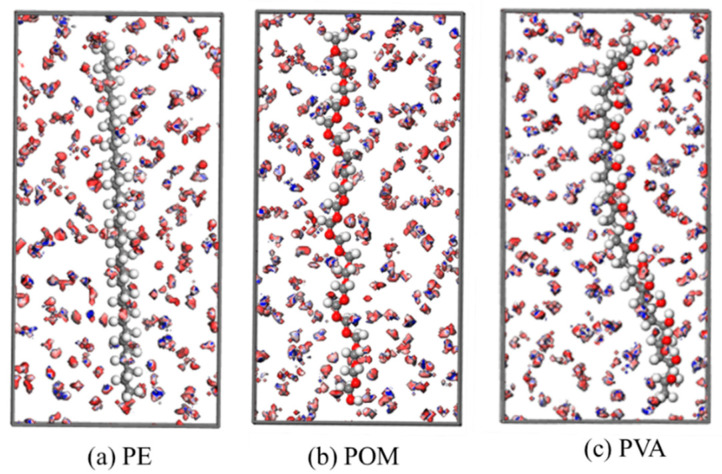
Representative isodensity surfaces colored with the potential energies for benzene adsorption on (**a**) PE, (**b**) POM, and (**c**) PVA obtained from GCMC simulations (The dark red and blue areas denote the highest and lowest energy, respectively).

As shown in Table S1 and Figure 2, the *C*_m_ values for 48 organic compounds towards 12 nanoplastics range from 1.58 to 150.80 g/g. Among these nanoplastics, polyethylene (PE), polyoxymethylene (POM), and polyvinyl alcohol (PVA) (Figure 3), exhibit *C*_m_ values within the top 30%, demonstrating their superior adsorption capability compared to the other nanoplastics examined. The *C*_m_ values (g/g) for PE are in the range of 5.60~150.80 g/g, while those for POM range from 15.90 to 55.66 g/g, and for PVA, from 4.99 to 43.75 g/g. To better understand the adsorptions on these three MPs, Figure 3 presents the isodensity surfaces for benzene adsorption, colored by potential energies, revealing distinct favorable binding sites for benzene on PE, POM and PVA within their respective polymer frameworks.

Furthermore, we computed the adsorption of 48 organic compounds on PE, POM, and PVA nanoplastics using DFT, and obtained the corresponding log*K* values. In our previous studies [58,59], we have validated the reliability of adsorption data from DFT computations. Besides, we compared the gas-phase log*K* values for five organic compounds (i.e., benzene, naphthalene, anthracene, nitrobenzene and phenol) on PE MPs from our DFT computations with those from molecular dynamics (MD) simulations [60], for which the methodology had been benchmarked against experimental aqueous log*K* data. These log*K* values from DFT computations agreed well with those from the verified MD simulations (Table S2). Thus, the atmospheric log*K* values obtained in this study (Table S1) can be considered as robust and reliable. The optimized adsorption equilibrium configurations for the 48 organic compounds on PE, POM, and PVA nanoplastics are presented in Table S3. The distances between the molecular mass centers and the nanoplastics range from 2.87 to 5.91 Å.

### 3.2. Sorbent-Specific QSAR Models for Predicting logK Values on PE, POM, and PVA Nanoplastics

The optimal prediction models for estimating the log*K* values on PE, POM, and PVA nanoplastics were as follows:

for PE nanoplastics,

log*K* = −10.626 + 2.445 × *ATSC0p* + 4.041 × *SpMax1_Bhm* − 1.261 × *LipoaffinityIndex*

*n_t_ =* 40, *R*^2^_adj_ = 0.95, *RMSE*_t_
*=* 2.32, *F* = 257.17, *p* < 0.001, (3)

*n*_v_ = 8, *R*^2^_ext_ = 0.92, *Q*^2^_ext_ = 0.91, *RMSE*_V_
*=* 3.09

for POM nanoplastics,

log*K* = −6.299 + 2.17 × *ATSC0p* + 3.453 × *SpMax1_Bhm* − 1.266 × *LipoaffinityIndex*

*n_t_ =* 40, *R*^2^_adj_ = 0.93, *RMSE*_t_
*=* 2.53, *F* = 163.77, *p* < 0.001, (4)

*n*_v_ = 8, *R*^2^_ext_ = 0.97, *Q*^2^_ext_ = 0.96, *RMSE*_V_
*=* 1.74

and for PVA nanoplastics,

log*K* = −9.963 + 2.879 × *ATSC0p* + 4.168 × *SpMax1_Bhm* − 2.246 × *LipoaffinityIndex*

*n_t_ =* 40, *R*^2^_adj_ = 0.91, *RMSE*_t_
*=* 3.57, *F* = 139.02, *p* < 0.001, (5)

*n*_v_ = 8, *R*^2^_ext_ = 0.94, *Q*^2^_ext_ = 0.91, *RMSE*_V_
*=* 3.23

**Figure 4:**
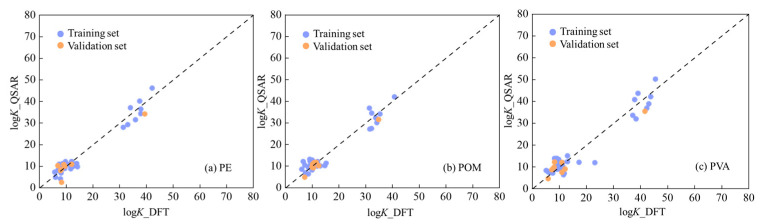
Predicted log*K* values with QSAR models (log*K___*QSAR) versus those from DFT calculations (log*K___*DFT).

Here, *n_t_* and *n*_v_ represent the number of organic compounds in the training set and validation set, respectively. To evaluate the goodness-of-fit, robustness and predictive capability, we calculated a few parameters, including *R*^2^_adj_, *R*^2^_ext_, *Q*^2^_ext_, *RMSE*_t_, and *RMSE*_V_. According to the established criteria (*R*^2^ > 0.60 and *Q*^2^ > 0.50) [61], these three QSAR models perform well. Additionally, the variable inflation factors (*VIF*) values for the predictive variables (Table S4) used in these three QSAR models are less than 10, indicating that no serious multi-collinearity exists among these variables [62]. Figure 4 compares the QSAR-predicted log*K* values with the DFT-calculated log*K* values for PE, POM, and PVA, demonstrating the predictive accuracy of the developed models. The close agreement indicates that these QSAR models provide a reliable and efficient alternative to additional simulations or experiments for rapidly estimating logK values of organic pollutants on PE, POM, and PVA nanoplastics.

Then, we characterized the application domains (ADs) of the developed QSAR models on the basis of δ* and *h*_i_ values (Figure S1). All standardized residuals for the organic compounds in both the training set and the validation set are in the range of −3 to 3, which implies that no outlier exists. Note that the leverage value for one compound in the validation, namely, cresyl diphenyl phosphate, exceeds the warning leverage value of 0.3, which suggests that the structure of cresyl diphenyl phosphate significantly differs from those of the organic compounds in the training set. Nevertheless, the predicted log*K* values for cresyl diphenyl phosphate on nanoplastics from the QSAR models align well with the DFT values, indicating that the established QSAR models possess reliable extrapolation capabilities. Consequently, these three QSAR models can reliably estimate adsorption on PE, POM, and PVA nanoplastics for a wide range of organic compounds, including alkanes, alkenes, aromatic hydrocarbons, alcohols, ketones, aldehydes, carboxylic acids, phenols, anilines, nitriles, nitrobenzenes, esters, polycyclic aromatic hydrocarbons, biphenyls, halogenated benzenes, polybrominated diphenyl ethers, and organophosphates.

### 3.3. Multi-Dimensional QSAR Models for Predicting C_m_ Values on Nanoplastics

Note that each of the aforementioned QSAR models for predicting log*K* values is specific to a particular nanoplastic. To predict the log*K* values on different nanoplastics, separate models must be established for each material. This limitation underscores the necessity of developing a multi-dimensional predictive model capable of efficiently generating adsorption data across diverse nanoplastics. Considering computational costs and data volume, we focused on predicting *C*_m_ values as an example to explore the development of a multi-dimensional prediction model.

Firstly, we developed 12 QSAR models using the *C*_m_ values and structural descriptors of 48 organic compounds. Afterward, a multi-dimensional prediction model was constructed by integrating these 12 QSAR models with the structural descriptors of the 12 nanoplastics.

#### 3.3.1. Twelve Sorbent-Specific QSAR Models

QSAR models for predicting the *C*_m_ values on 12 nanoplastics are shown below.

For PA nanoplastics,

*C*_m_ = 8.108 − 0.017 × *AATS5m* + 0.395 × *MATS8c* + 0.0446 × *GATS8c* − 4.639 × *VE1_DzZ* + 1.556 × *AMW*
*n =* 48, *R*^2^_adj_ = 0.94, *RMSE =* 1.27, *F* = 148.69, *p* < 0.001 (6)

For PC nanoplastics,

*C*_m_ = 6.706 − 0.014 × *AATS5m* + 0.043 × *MATS8c* + 0.456 × *GATS8c* − 3.686 × *VE1_DzZ* + 1.289 × *AMW*
*n =* 48, *R*^2^_adj_ = 0.94, *RMSE =* 1.05, *F* = 149.46, *p* < 0.001 (7)

For PE nanoplastics,

*C*_m_ = 12.592 − 0.018 × *AATS5m* + 75.837 × *MATS8c* − 25.79 × *GATS8c* − 46.757 × *VE1_DzZ* + 2.86 × *AMW* *n =* 48, *R*^2^_adj_ = 0.79, *RMSE =* 8.58, *F* = 36.01, *p* < 0.001 (8)

For PP nanoplastics,

*C*_m_ = 8.226 − 0.017 × *AATS5m* + 0.511 × *MATS8c* + 0.559 × *GATS8c* − 7.509 × *VE1_DzZ* + 1.551 × *AMW* *n =* 48, *R*^2^_adj_ = 0.88, *RMSE =* 1.86, *F* = 71.33, *p* < 0.001 (9)

For PS nanoplastics,

*C*_m_ = 2.647 − 0.006 × *AATS5m* + 0.145 × *MATS8c* + 0.196 × *GATS8c* − 1.06 × *VE1_DzZ* + 0.52 × *AMW*
*n =* 48, *R*^2^_adj_ = 0.77, *RMSE =* 0.89, *F* = 31.95, *p* < 0.001 (10)

For PU nanoplastics,

*C*_m_ = 4.904 − 0.010 × *AATS5m* + 0.09 × *MATS8c* + 0.216 × *GATS8c* − 2.58 × *VE1_DzZ* + 0.922 × *AMW*
*n =* 48, *R*^2^_adj_ = 0.94, *RMSE =* 0.56, *F* = 150.16, *p* < 0.001 (11)

For PAA nanoplastics,

*C*_m_ = 4.718 − 0.01 × *AATS5m* + 0.082 × *MATS8c* + 0.183 × *GATS8c* − 2.394 × *VE1_DzZ* + 0.9 × *AMW*
*n =* 48, *R*^2^_adj_ = 0.95, *RMSE =* 0.68, *F* = 172.98, *p* < 0.001 (12)

For PET nanoplastics,

*C*_m_ = 4.317 − 0.011 × *AATS5m* + 0.143 × *MATS8c* + 0.213 × *GATS8c* − 2.491 × *VE1_DzZ* + 0.849 × *AMW*
*n =* 48, *R*^2^_adj_ = 0.94, *RMSE =* 0.64, *F* = 153.35, *p* < 0.001 (13)

For POM nanoplastics,

*C*_m_ = 10.747 − 0.019 × *AATS5m* + 0.3 × *MATS8c* + 0.543 × *GATS8c* − 7.442 × *VE1_DzZ* + 1.99 × *AMW*
*n =* 48, *R*^2^_adj_ = 0.93, *RMSE =* 1.88, *F* = 122.73, *p* < 0.001 (14)

For PVC nanoplastics,

*C*_m_ = 6.189 − 0.013 × *AATS5m* + 0.093 × *MATS8c* + 0.384 × *GATS8c* − 3.872 × *VE1_DzZ* + 1.174 × *AMW*
*n =* 48, *R*^2^_adj_ = 0.95, *RMSE =* 0.85, *F* = 166.24, *p* < 0.001 (15)

For PVA nanoplastics,

*C*_m_ = 8.694 − 0.018 × *AATS5m* + 0.739 × *MATS8c* + 0.682 × *GATS8c −* 10.196 × *VE1_DzZ* + 1.676 × *AMW*
*n =* 48, *R*^2^_adj_ = 0.84, *RMSE =* 2.44, *F* = 48.61, *p* < 0.001 (16)

For PMMA nanoplastics,

*C*_m_ = 4.009 − 0.01 × *AATS5m* + 0.174 × *MATS8c* + 0.242 × *GATS8c* − 2.58 × *VE1_DzZ* + 0.825 × *AMW*
*n =* 48, *R*^2^_adj_ = 0.95, *RMSE =* 0.62, *F* = 165.85, *p* < 0.001 (17)

**Figure 5:**
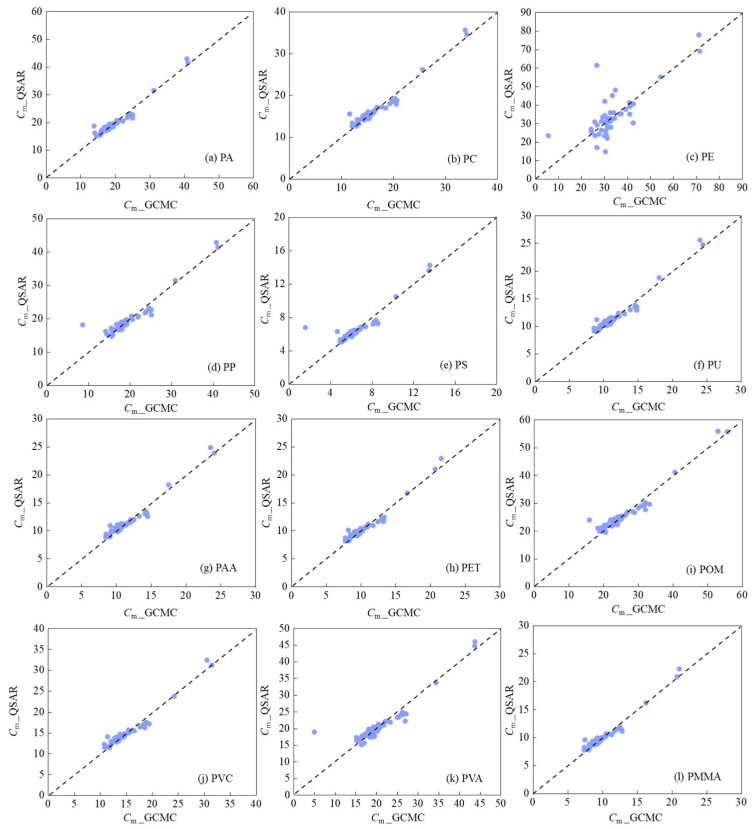
Predicted *C*_m_ values on (**a**) PA, (**b**) PC, (**c**) PE, (**d**) PP, (**e**) PS, (**f**) PU, (**g**) PAA, (**h**) PET, (**i**) POM, (**j**) PVC, (**k**) PVA and (**l**) PMMA with QSAR models (*C*_m*_*_QSAR) versus those from GCMC simulations (*C*_m*_*_GCMC).

All 12 QSAR models achieved *R*^2^_adj_ values exceeding the criterion of *R*^2^ > 0.60, indicating their strong goodness-of-fit. Moreover, no serious multi-collinearity was observed among the five variables, as their *VIF* values were below than 10 (Table S4). As shown in Figure 5, the predicted *C*_m_ values by the established QSAR models agree well with those obtained from GCMC simulations, further validating the performance of the models.

As illustrated in Figures S2 and S3, we also evaluated the ADs of the 12 QSAR models for *C*_m_ values using the δ* and *h* values. Note that for these 12 QSAR models, the *h* values for fluorene (0.78) and heptabromodiphenyl ether (0.91) exceeded the warning leverage value h* (0.375), while their δ* values remained within the range of −3~3. These results indicate that the structures of fluorene and heptabromodiphenyl ether are significantly different from other compounds in the dataset, and they are influential on the QSAR models.

Additionally, some outliers were identified based on |δ*| values (larger than 3): for the QSAR models of *C*_m_ values on PA, PC, PP, POM, PVA, and PMMA nanoplastics, formaldehyde is an outlier. For the QSAR model on PE, n-propylbenzene is an outlier. Likewise, 4-fluorophenol is an outlier for the QSAR model on PS nanoplastics.

In general, the AD of a prediction model depends on the dataset used. Since all 12 QSAR models were developed with the same dataset consisting of 48 different organic compounds, they share the same ADs covering a wide range of compounds with diverse functional groups, including -OH, -NH_2_, -NO_2_, -CH_3_, -CN, -CHO, -CH_2_OH, -CH_2_CH_3_, -C(O)CH_3_, -CH_2_CH_2_OH, -OC(O)CH_3_, -C(O)OCH_3_, -CH_2_CH_2_CH_3_, -C(O)OCH_2_CH_3_, -F, -Br, OP-(OR)_3_, and -C_6_H_5_.

#### 3.3.2. A Multi-Dimensional Prediction Model

Both the QSAR models for log*K* values and those for *C*_m_ values developed above are specific to individual nanoplastics, as each model solely employs molecular structural descriptors of organic compounds as input features. These descriptors cannot capture the structural characteristics of the nanoplastics themselves, and thus are limited to single adsorbents. To overcome this limitation, we incorporated structural descriptors of the nanoplastics into the QSAR models, aiming at developing a multi-dimensional prediction model that estimate the adsorption of various organic compounds on a diverse range of nanoplastics.

Based on the 12 sorbent-specific QSAR models developed for predicting *C*_m_ values, we derived a simplified model to unify their predictive capabilities into a single framework:

*C*_m_ = *c* + *x*_1_ × *AATS5m* + *x*_2_ × *MATS8c* + *x*_3_ × *GATS8c* + *x*_4_ × *VE1_DzZ* + *x*_5_ × *AMW* (18)

where *AATS5m*, *MATS8c*, *GATS8c*, *VE1_DzZ*, and *AMW* are descriptors for the organic compounds. Recognizing that the adsorption is influenced not only by the structures of the organic compounds but also by the characteristics of nanoplastics, we made an attempt to incorporate some descriptors reflecting the structural features of nanoplastics. This approach allows us to predict the constant *c* and other coefficients (*x*_1_, *x*_2_, *x*_3_, *x*_4_, and *x*_5_) in Equation (18), resulting in a prediction model with variables combining the structural information from both organic compounds and nanoplastics. The values for *c*, *x*_1_, *x*_2_, *x*_3_, *x*_4_, and *x*_5_ can be estimated with the following equations.

*c* = 11.361 − 5.839 × *GATS3v*, *r* = 0.889 (19)

*x*_1_ = −0.019 + 0.008 × *GATS3v*, *r* = 0.787 (20)

*x*_2_ = 71.408 − 92.984 × *SIC2*_,_
*r* = 0.755 (21)

*x*_3_ = −24.086 + 31.432 × *SIC2*, *r* = 0.749 (22)

*x*_4_ = −36.628 + 46.548 × *BIC3*, *r* = 0.798 (23)

*x*_5_ = 2.305 − 1.242 × *GATS3v*, *r* = 0.866 (24)

Accordingly, the optimal multi-dimensional prediction model is shown below.

*C*_m_ = (11.361 − 5.839 × *GATS3v*) + (−0.019 + 0.008 × *GATS3v*) × *AATS5m* + (71.408 − 92.984 × *SIC2*) × *MATS8c* + (−24.086 + 31.432 × *SIC2*) × *GATS8c* + (−36.628 + 46.548 × *BIC3*) × *VE1_DzZ* + (2.305 − 1.242 × *GATS3v*) × *AMW* (25)

Note that the values of *x*_2_ and *x*_3_ for PE differ significantly from those for other nanoplastics (Figure S4), making them influential in the univariate predictive model (Equations (21) and (22)). This observation highlights the need for future investigation into adsorption on a broader range of nanoplastics to expand the dataset. By incorporating more diverse nanoplastics and utilizing additional descriptors to better characterize their structural features, the robustness of the multi-dimensional prediction model can be enhanced, and its applicability domains (ADs) can be further expanded.

### 3.4. Adsorption Mechanisms

**Figure 6:**
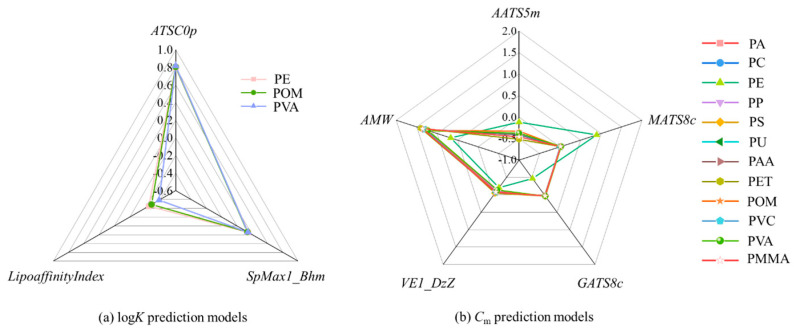
Radar compass plot with the standardized coefficients of (**a**) three descriptors in the log*K* prediction models and (**b**) five descriptors in the *C*_m_ prediction models.

As presented in Equations (3)–(16) and Table S5, the descriptors utilized in the developed QSAR models capture various structural characteristics. To compare the contributions of these descriptors, we used radar compass plots (Figure 6) with standardized coefficients. Besides, the details for the standardized coefficients, *t*, *p* values, and *VIF* values of these descriptors, are given in Table S4. Note that the descriptors used for predicting the log*K* values are different from those for predicting the *C*_m_ values (Table S4), suggesting that the factors influencing log*K* are different from those influencing *C*_m_. This distinction offers us valuable insights into the underlying adsorption mechanisms driving adsorption on nanoplastics.

#### 3.4.1. QSAR Models for log*K* Values

As shown in Figure 6a, the standardized coefficients of three descriptors, *ATSC0p*, *SpMax1_Bhm*, and *LipoaffinityIndex*, in the QSAR models (Equations (3)–(5)) for predicting the log*K* values on PE, POM, and PVA nanoplastics are significantly different from each other. This variation implies that these descriptors exert distinct influences on adsorption, reflecting the unique interactions between the nanoplastics and the organic pollutants.

For the adsorption on PE, POM, and PVA nanoplastics, the standardized coefficients of *ATSC0p* are significantly higher than those of *SpMax1_Bhm* and *LipoaffinityIndex* [Figure 6a]. It indicates that *ATSC0p* is the most influential predictive variable in predicting the log*K* values for organic compounds on PE, POM and PVA nanoplastics. The positive standardized coefficients of *ATSC0p* suggest that an increase in this descriptor for an organic compound can lead to an increase in the log*K* values.

*ATSC0p* [63] represents the centered Broto–Moreau autocorrelation-lag0/weighted by polarizabilities. It means that the polarizabilities of organic compounds can influence their adsorption on nanoplastics. Note that compounds with higher polarizability tend to have larger *ATSC0p* values. For example, benzene has a polarizability of 10.32, lower than that of cyclohexane (11.0) [64], and a correspondingly smaller *ATSC0p* value (3.02 for benzene vs. 4.01 for cyclohexane). Accordingly, the log*K* values for benzene on PE (5.74) and POM (6.13) are lower than those for cyclohexane (6.94 on PE and 8.62 on POM). However, for the adsorption on PVA, the log*K* value for benzene (5.76) is slightly higher than that for cyclohexane (5.13). This trend suggests that compounds with higher polarizability are more prone to be adsorbed by PE and POM nanoplastics, which may be attributed to stronger induced and dispersion forces between compounds with higher polarizability and these nanoplastics, leading to higher log*K* values on the nanoplastics.

Additionally, the standardized coefficients of *SpMax1_Bhm* in the QSAR models for predicting the log*K* values on PE, POM, and PVA nanoplastics are all positive, while those of *LipoaffinityIndex* are negative. Thus, the log*K* values can increase with increasing *SpMax1_Bhm* values for the organic compounds, while a decrease in *LipoaffinityIndex* also leads to higher log*K* values.

*SpMax1_Bhm* [63] represents the largest absolute eigenvalue of the Burden modified matrix-n1 weighted by relative mass. In general, molecules with larger mass tend to have higher molar volumes, and consequently more contact with nanoplastics, thereby having stronger van der Waals interactions. In contrast, the descriptor *LipoaffinityIndex* [65], which measures the lipoaffinity of an organic compound, is negatively correlated with log*K* values. Compounds having functional groups such as -OH, -NO_2_, and -NH_2_ typically have lower *LipoaffinityIndex* values, making them more readily adsorbed by nanoplastics. This may be because these functional groups can enhance dipole-induced dipole interactions between the organic pollutants and nanoplastics, thereby increasing the log*K* values.

#### 3.4.2. Sorbent-Specific QSAR Models for C_m_ Values

As shown in Equations (6)–(17), five descriptors, namely, *AATS5m*, *MATS8c*, *GATS8c*, *VE1_DzZ*, and *AMW*, are used in combination to predict the *C*_m_ values on 12 nanoplastics (PA, PC, PE, PP, PS, PU, PAA, PET, POM, PVC, PVA, and PMMA). Figure 6b illustrates that the standardized coefficients for these five descriptors vary in magnitude, highlighting their different contributions to predicting the *C*_m_ values.

Among these five descriptors, *AMW* exhibits the highest standardized coefficients in the QSAR models for predicting *C*_m_ values on all nanoplastics except PE, indicating that *AMW* is the most influential parameter in these predictions. *AMW* [66] denotes the average molecular weight (molecular weight divided by the total number of atoms). The positive standardized coefficients for *AMW* suggest that higher *C*_m_ values are associated with increased *AMW* values of organic compounds. In contrast, the standardized coefficients for *AATS5m* are negative, which implies that the *C*_m_ values increase as *AATS5m* decreases. *AATS5m* [67] represents average Broto–Moreau autocorrelation-lag5 weighted by mass, which is also related to the molecular weight. These findings highlight the critical role of molecular weight, as characterized by *AMW* and *AATS5m*, in predicting the *C*_m_ values.

Additionally, the standardized coefficients for *MATS8c* and *GATS8c* are positive in the QSAR models for predicting *C*_m_ values on all nanoplastics except PE, which indicates that the *C*_m_ values increase with higher *MATS8c* and *GATS8c* values. *MATS8c* [68] is Moran autocorrelation-lag8 weighted by charges, while *GATS8c* [69] represents Geary autocorrelation-lag8 weighted by charges. These two descriptors capture charge-related interactions well, suggesting that compounds with higher charges (reflected by higher *MATS8c* and *GATS8c* values) exhibit stronger electrostatic interactions with nanoplastics, thus leading to higher *C*_m_ values.

Lastly, the descriptor *VE1_DzZ* [63], which represents the coefficient sum of the last eigenvector from Barysz matrix weighted by atomic number, has negative standardized coefficients, which indicates that *VE1_DzZ* contributes negatively to the *C*_m_ values.

Notably, on PE nanoplastics, the standardized coefficient of *GATS8c* for predicting the *C*_m_ values is negative, in contrast to its positive correlation on the other 11 nanoplastics. The negative coefficient indicates that, for PE, *GATS8c* is negatively correlated with *C*_m_ values. For PE nanoplastics, *MATS8c* has the highest standardized coefficient, indicating that it is the most influential parameter among these five descriptors in predicting *C*_m_ values. Similar to their role in the other 11 nanoplastics, *GATS8c* and *MATS8c* combinedly reflect charge-related interactions, which contributes to the prediction of *C*_m_ values on PE nanoplastics.

#### 3.4.3. Multi-Dimensional QSAR Models for *C*_m_ Values

According to the multi-dimensional prediction model (Equation (25)), three additional descriptors, namely, *GATS3v*, *SIC2*, and *BIC3*, characterizing the structural properties of the nanoplastics, also influence *C*_m_ values.

*GATS3v* [70] represents Geary autocorrelation-lag3 weighted by van der Waals volumes, highlighting the significant role of van der Waals interactions in the adsorption of organic compounds on nanoplastics. It is consistent with the adsorption mechanisms revealed by the QSAR models for predicting log*K* values.

In addition, the descriptors *SIC2* and *BIC3* are also related to the prediction of *C*_m_ values. *SIC2* [63] denotes structural information content index (neighborhood symmetry of 2-order), while *BIC3* [71] is bond information content index (neighborhood symmetry of 3-order).

#### 3.4.4. Effects of Functional Groups on Adsorption Energies

To explore the effects of functional groups on adsorption, we compared the adsorption energies of compounds with different functional groups on PE, POM, and PVA nanoplastics (Table S6). Note that the adsorption energies for benzene on PE (−7.36 kcal/mol) and POM (−8.54 kcal/mol) are more negative than those for cyclohexane on PE (−6.37 kcal/mol) and POM (−8.18 kcal/mol). This difference may arise from the π electrons in benzene, which enhance the electrostatic interactions with PE and POM nanoplastics. In contrast, the adsorption energy for benzene on PVA (−7.44 kcal/mol) is higher than that for cyclohexane (−8.52 kcal/mol), likely due to repulsive interactions between the π electrons of benzene and the electrons of -OH group of PVA, which reduce the adsorption.

For benzene derivatives with mono-substituent, the interactions with PE/POM/PVA nanoplastics are generally stronger than those with benzene, irrespective of the substituent’s electrophilic properties. For example, benzyl alcohol, with a substituent (-CH_2_OH) that has a Hammett sigma meta parameter of zero (*σ*_m_ = 0), exhibits stronger adsorption energies on PE (−9.79 kcal/mol), POM (−14.81 kcal/mol), and PVA (−16.11 kcal/mol) compared to benzene on these nanoplastics (PE, −7.36 kcal/mol; POM, −8.54 kcal/mol; PVA, −7.44 kcal/mol). The enhanced adsorption can be ascribed to the dispersion interactions between the substituents and the nanoplastics, which promote stronger binding of benzene derivatives.

Notably, nitrobenzene exhibits slightly weaker adsorption energy on POM compared to benzene, which could be due to electrostatic repulsion between the electron-withdrawing -NO_2_ group on nitrobenzene and the electron-rich oxygen atoms in POM, weakening the adsorption.

## 4. Conclusions

Based on GCMC simulations, PE, POM, and PVA nanoplastics exhibit the highest adsorption capacities among the twelve nanoplastics investigated for the 39 representative aliphatic and aromatic compounds and the nine emerging pollutants (brominated flame retardants and phosphorus flame retardants). The QSAR analysis further reveals that *ATSC0p*, a descriptor associated with molecular polarizability, is the most influential factor governing log*K* values on PE, POM, and PVA, underscoring the key role of dispersion-related effects in pollutant uptake. For adsorption capacities (*C*_m_), a set of QSAR models was established for all twelve nanoplastics, and an integrated multi-dimensional prediction model was further developed to enable rapid, one-step screening of adsorption capacities for diverse pollutants across multiple nanoplastic types.

Mechanistically, all these developed models consistently indicate that van der Waals interactions, supplemented by electrostatic interactions for more polar compounds, dominate adsorption of organic compounds on nanoplastics under gas-phase conditions relevant to atmospheric environments. Collectively, the proposed GCMC-DFT-QSAR framework and the multi-dimensional predictive model substantially improve the efficiency of generating adsorption data for pollutant–nanoplastic pairs, providing a practical basis for screening environmental behavior and supporting ecological risk assessment of organic pollutants associated with atmospheric nanoplastics.

## Data Availability

The data supporting this article have been included in the Supplementary Information.

## Supplementary Materials

The following supporting information can be downloaded at: https://www.mdpi.com/article/10.3390/nano16030178/s1, Table S1: Organic compounds and estimated adsorption capacity (*C*_m_, g/g) from GCMC simulations and logarithm values for adsorption equilibrium coefficient (log*K*) from DFT computations under atmospheric conditions; Table S2: The atmospheric log*K* values for five organic compounds on PE NPs from DFT computations and those from MD simulations; Table S3: Adsorption equilibrium configuration for the 48 organic compounds towards PE, POM and PVA nanoplastics; Table S4: Standardized coefficients, *t*, *p* values and variable inflation factor (*VIF*) for the predictive variables; Table S5: Definitions for the descriptors utilized in the developed models; Table S6: Hammett parameters of substituents for 10 different organic compounds and their adsorption energies (*E*_ad_) on PE, POM and PVA nanoplastics; Figure S1: Application domain characterized by Williams plots with standardized residuals (*δ**) and leverage values (*h*) for QSAR models of log*K* values on (a) PE, (b) POM and (c) PVA nanoplastics (*h**: the warning leverage value); Figure S2: Application domain characterized by Williams plots with standardized residuals (*δ**) and leverage values (*h*) for QSAR models of *C*_m_ values on (a) PA, (b) PC, (c) PE, (d) PP, (e) PS and (f) PU nanoplastics (*h**: the warning leverage value); Figure S3: Application domain characterized by Williams plots with standardized residuals (*δ**) and leverage values (*h*) for QSAR models of *C*_m_ values on (a) PAA, (b) PET, (c) POM, (d) PVC, (e) PVA and (f) PMMA nanoplastics (*h**: the warning leverage value); Figure S4: Prediction for c, x_1_, x_2_, x_3_, x_4_ and x_5_ based on descriptors characterizing nanoplastics.
