# Supplementary material for: In Silico Models for Predicting Adsorption of Organic Pollutants on Atmospheric Nanoplastics by Combining Grand Canonical Monte Carlo/Density Functional Theory and Quantitative Structure Activity Relationship Approach
Source: Nanomaterials (Basel). 2026 Jan 28;16(3):178. doi: 10.3390/nano16030178 (PMC12899699; doi:10.3390/nano16030178)
Supplement: Supplementary file 1 [file nanomaterials-16-00178-s001.zip › nanomaterials-4075551-supplementary.pdf]

In silico Models for Predicting Adsorption of Organic Pollutants on Atmospheric Nanoplastics by Combining Grand Canonical Monte Carlo/Density Functional Theory and Quantitative Structure Activity Relationship Approach

Ya Wang,[1] Honghong Yi,[1] Chao Li,[2,*] Xiaolong Tang,[1] Peng Zhao,[1] Zhongfang Chen,[3,*]

[1] School of Energy and Environmental Engineering, University of Science and Technology Beijing, Beijing 10083, China

[2] Engineering Laboratory for Water Pollution Control and Resources Recovery, State Environmental Protection Key Laboratory of Wetland Ecology and Vegetation Restoration, School of Environment, Northeast Normal University, Changchun, 130117, China

[3] Department of Chemistry, University of Puerto Rico, San Juan, PR 00931, USA

Corresponding Authors

* Chao Li, e-mail: lic932@nenu.edu.cn (CL)

* Zhongfang Chen, e-mail: zhongfang.chen1@upr.edu (ZC)

The supplementary material consists of six tables and four figures. There are 22 pages in total.

Contents

Table S1. Organic compounds and estimated adsorption capacity (C_m, g/g) from GCMC simulations and logarithm values for adsorption equilibrium coefficient (log*K*) from DFT computations under atmospheric conditions 3

Table S2. The atmospheric log*K* values for five organic compounds on PE NPs from DFT computations and those from MD simulations 7

Table S3. Adsorption equilibrium configuration for the 48 organic compounds towards PE, POM and PVA nanoplastics 8

Table S4. Standardized coefficients, *t*, *p* values and variable inflation factor (*VIF*) for the predictive variables 15

Table S5. Definitions for the descriptors utilized in the developed models 17

Table S6. Hammett parameters of substituents for 10 different organic compounds and their adsorption energies (E_{ad}) on PE, POM and PVA nanoplastics........ 18

Figure S1. Application domain characterized by Williams plots with standardized residuals (δ*) and leverage values (*h*) for QSAR models of log*K* values on (a) PE, (b) POM and (c) PVA nanoplastics (*h**: the warning leverage value) 19

Figure S2. Application domain characterized by Williams plots with standardized residuals (δ*) and leverage values (*h*) for QSAR models of C_m values on (a) PA, (b) PC, (c) PE, (d) PP, (e) PS and (f) PU nanoplastics (*h**: the warning leverage value) 20

Figure S3. Application domain characterized by Williams plots with standardized residuals (δ*) and leverage values (*h*) for QSAR models of C_m values on (a) PAA, (b) PET, (c) POM, (d) PVC, (e) PVA and (f) PMMA nanoplastics (*h**: the warning leverage value) 21

Figure S4. Prediction for *c*, x_1, x_2, x_3, x_4 and x_5 based on descriptors characterizing nanoplastics (*r* is the correlation coefficient) 22

Table S1. Organic compounds and estimated adsorption capacity (C_m, g/g) from GCMC simulations and logarithm values for adsorption equilibrium coefficient (log$K$) from DFT computations under atmospheric conditions

No.	Compound	Adsorption capacity (C_m)												log$K$		
		PA	PC	PE	PP	PS	PU	PAA	PET	POM	PVC	PVA	PMMA	PE	POM	PVA
1	formaldehyde	13.83	11.57	5.6	8.6	4.66	8.98	9.11	8.18	15.9	11.4	4.99	7.48	8.29	7.21	5.89
2	formic acid	24.99	20.52	42.46	25.15	8.56	14.81	14.49	13.21	32.05	18.55	26.9	12.82	7.91	8.58	11.81
3	malonic acid	23.78	20.34	41.07	24.72	8.19	14.55	14.1	12.97	31.18	18.33	26.34	12.4	8.14	14.76	17.28
4	acetaldehyde	15.97	13.03	25.85	15.49	5.4	9.64	9.26	8.51	20.22	12.11	16.41	8.1	6.03	7.48	7.56
5	isoprene	14.07	12.04	24.17	14.16	4.94	8.6	8.5	7.74	18.32	10.77	15.14	7.4	8.1	10.01	6.11
6	cyclohexane	14.74	12.27	24.21	14.44	5.09	8.63	8.52	7.88	19.2	11.21	15.79	7.49	6.94	8.62	5.13
7	methylcyclohexane	14.58	12.06	24.46	14.69	4.93	8.6	8.47	7.72	18.84	10.78	15.21	7.36	8.03	8.98	9.11
8	benzene	16.76	14.11	28.75	16.7	5.66	10.08	9.8	9.04	21.88	12.76	18	8.58	5.74	6.13	5.76
9	phenol	19.64	16.18	32.7	19.32	6.57	11.55	11.42	10.28	25.3	14.71	20.8	10.05	9.03	13.05	10.77
10	benzonitrile	18.25	15.12	30.89	18.62	6.21	11.07	10.66	9.94	24.09	13.82	19.72	9.27	7.91	10.5	12.15

11	nitrobenzene	21.2	17.78	37.22	21.99	7.37	12.95	12.52	11.13	28.45	16.18	22.79	10.91	8.39	7.25	10.17
12	aniline	18.31	15	31.31	18.47	6.24	11.03	10.68	9.75	23.97	13.69	19.71	9.57	8.55	10.4	8.35
13	toluene	16.46	13.4	27.57	16.31	5.55	9.88	9.49	8.81	21.16	12.44	17.06	8.41	8.16	9.93	7.24
14	benzyl alcohol	18.05	15.19	31.2	18.11	6.09	10.98	10.5	9.7	23.98	13.54	19.48	9.25	8.17	11.82	10.46
15	acetophenone	18.4	15.49	30.55	18.09	6.22	10.93	10.58	9.78	23.78	13.7	19.49	9.26	8.65	11.02	9.43
16	ethylbenzene	15.89	13.35	26.71	15.89	5.42	9.43	8.98	8.42	20.37	11.88	17.06	8.13	9.3	10.72	9.3
17	ethyl benzoate	17.77	15.34	30.94	17.84	6.1	10.7	10.31	9.76	23.59	13.34	19.48	9.28	9.74	10	9.79
18	phenethyl alcohol	17.43	14.46	28.7	17.56	5.76	10.25	10.17	9.27	22.29	13.13	18.31	8.69	8.61	11.88	9.83
19	phenyl acetate	19.43	15.51	32.29	18.6	6.47	11.14	10.86	10	24.47	14.15	20.06	9.63	9.13	10.77	10.96
20	methyl benzoate	19.27	15.65	32.56	19.26	6.38	11.43	10.95	10.24	24.89	14.51	20.41	9.73	12.06	11.5	8.24
21	n-propylbenzene	15.62	12.87	26.72	15.56	5.25	9.15	9.09	8.28	20.52	11.77	16.35	8.03	6.87	10.19	8.03
22	4-fluorophenol	22.24	18.5	36.55	21.97	1.58	13.02	12.44	11.73	28.9	16.53	23.3	11.34	11.71	11.91	11.38
23	m-cresol	18.6	15.3	31.33	18.64	6.19	11.21	10.65	9.78	23.85	13.64	19.52	9.5	8.83	9.51	9.77
24	1,2-dinitrobenzene	25.02	20.66	42.53	25.16	8.44	14.82	14.39	13.23	33.27	19.41	27.18	12.57	8.86	9.27	9.09

25	1,3-dinitrobenzene	23.74	19.82	42.01	24.95	8.37	14.7	14.18	13.23	31.74	18.51	25.91	12.71	11.23	11.99	8.25
26	1,4-dinitrobenzene	24.38	20.29	40.86	24.56	8.37	14.58	14.25	13.34	31.97	18.85	26.25	12.49	7.39	6.67	8.99
27	4-nitrotoluene	20.52	16.44	34.42	20.6	6.83	12.25	11.98	10.87	26.31	15.27	21.76	10.44	10.03	9.79	23.12
28	p-xylene	15.92	13.14	26.8	15.51	5.38	9.5	9.35	8.48	20.85	12.07	17.22	8.18	7.39	9.86	9.79
29	4-ethylphenol	18	14.81	30.42	17.98	5.92	10.51	10.25	9.53	22.89	13.24	18.92	9.06	9.43	9.99	11.29
30	3-methylbenzyl alcohol	17.16	14.4	29.94	17.17	6.04	10.51	10.08	9.51	22.59	13.19	18.77	8.86	13.96	10.43	13.02
31	2,4-dinitrotoluene	23.45	19.27	39.23	23.65	8.09	13.87	13.28	12.43	29.99	17.63	25.02	11.71	12.01	9.1	13.01
32	3,5-dimethylphenol	17.57	14.52	30.18	17.85	6.08	10.58	10.25	9.58	23.25	13.34	19.24	9	8.8	10.89	10.36
33	biphenyl	18.25	14.85	30.17	17.95	6.26	10.77	10.35	9.73	23.06	13.69	19.23	9.38	9.12	10.71	9.62
34	naphthalene	18.59	14.94	31.41	18.11	6.21	10.76	10.75	10	24.2	13.66	19.62	9.52	9.49	10.51	7.47
35	1-methylnaphthalene	17.98	15.04	30.91	18.41	6.14	10.53	10.65	9.8	23.41	13.92	19.52	9.41	9.92	12.31	7.44
36	fluorene	19.09	15.51	150.8	19.08	6.31	11.36	10.95	10.24	24.38	14.17	20.5	9.74	13.36	15.21	10.24
37	anthracene	19.02	16.11	32.76	19.18	6.46	11.56	11	10.36	25.04	14.72	20.65	10.08	12.59	11.92	11.11
38	phenanthrene	19.23	16.28	33.46	19.14	6.62	11.69	11.19	10.43	25.58	14.45	20.43	9.9	9.53	12.47	10.94

39	pyrene	20.46	16.49	34.77	20.38	6.87	12.09	11.69	10.65	26.42	15.63	21.91	10.48	14.23	10.85	11.54
40	(1-bromoethyl)benzene	23.83	19.63	40.96	23.96	8.32	14.74	14.15	13.07	30.85	18.24	25.4	12.24	33.05	32.19	38.33
41	2,4,6-tribromophenol	40.73	33.79	71.04	40.79	13.56	24.03	23.51	21.53	52.97	30.51	43.75	21.06	37.93	35.35	43.75
42	2,4,4'-tribromodiphenyl ether	31.15	25.58	54.4	30.93	10.31	18.08	17.49	16.67	40.57	24.21	34.32	16.26	37.51	31.39	39.06
43	heptabromodiphenyl ether	41.1	34.07	71.39	41.19	13.47	24.46	24.05	20.67	55.66	31.44	43.68	20.75	42.17	40.76	45.49
44	trimethyl phosphate	20.37	16.83	34.02	20.34	6.92	12.14	12.04	11.02	26.92	15.42	22.31	10.49	31.38	31.53	37.11
45	triethyl phosphate	17.33	14.37	30.24	17.52	6.01	10.28	10.01	9.24	22.01	13.1	18.12	8.87	35.93	34.2	42.31
46	triisopropyl phosphate	17.15	13.98	29.25	17.31	5.68	10.15	9.95	9.33	23.02	13.28	18.5	8.68	37.89	33.73	43.06
47	triisobutyl phosphate	15.76	13.08	25.79	15.81	5.23	9.2	8.83	8.31	19.39	12.06	16.88	8.15	34.03	32.25	37.83
48	cresyl diphenyl phosphate	18.53	15.04	30.68	18.41	5.94	10.79	10.15	9.74	24.16	14.12	19.72	9.13	39.41	34.86	41.61

Table S2. The atmospheric log$K$ values for five organic compounds on PE NPs from DFT computations and those from MD simulations

No.	Compound	log$K$	
		DFT	MD (D = 100 Å) (Su et al. 2024)
1	benzene	5.74	5.94
2	naphthalene	9.49	8.50
3	anthracene	12.59	11.56
4	nitrobenzene	8.39	8.17
5	phenol	9.03	8.74

Table S3. Adsorption equilibrium configuration for the 48 organic compounds towards PE, POM and PVA nanoplastics

PE					
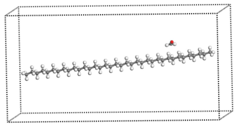<br>*d* = 3.73 Å<br>formaldehyde	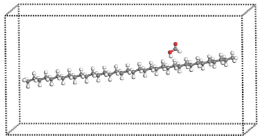<br>*d* = 4.05 Å<br>formic acid	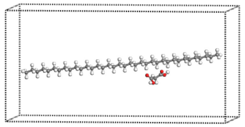<br>*d* = 3.74 Å<br>malonic acid	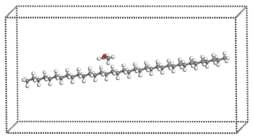<br>*d* = 2.87 Å<br>acetaldehyde	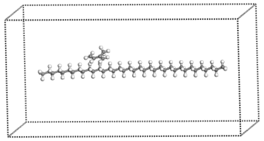<br>*d* = 3.74 Å<br>isoprene	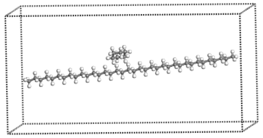<br>*d* = 3.93 Å<br>cyclohexane
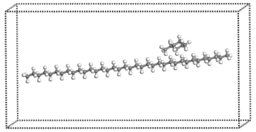<br>*d* = 3.99 Å<br>methylcyclohexane	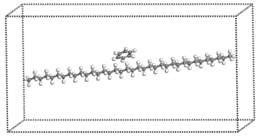<br>*d* = 3.38 Å<br>benzene	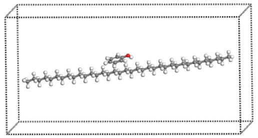<br>*d* = 3.37 Å<br>phenol	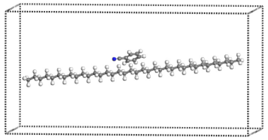<br>*d* = 3.53 Å<br>benzonitrile	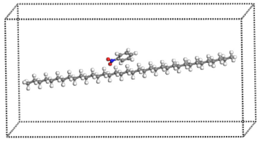<br>*d* = 3.41 Å<br>nitrobenzene	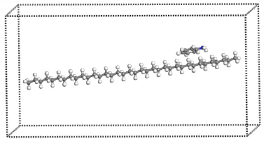<br>*d* = 3.35 Å<br>aniline
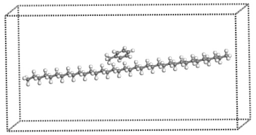<br>*d* = 3.37 Å<br>toluene	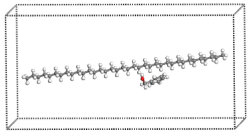<br>*d* = 3.79 Å<br>benzyl alcohol	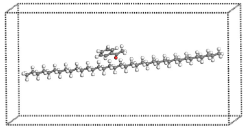<br>*d* = 3.43 Å<br>acetophenone	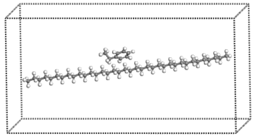<br>*d* = 3.76 Å<br>ethylbenzene	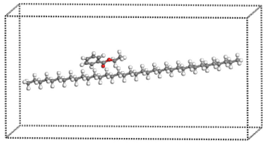<br>*d* = 3.42 Å<br>ethyl benzoate	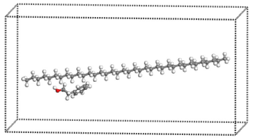<br>*d* = 3.87 Å<br>phenethyl alcohol

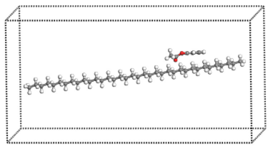<br>*d* = 3.64 Å<br>phenyl acetate	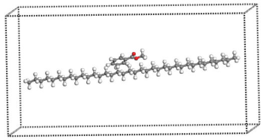<br>*d* = 3.47 Å<br>methyl benzoate	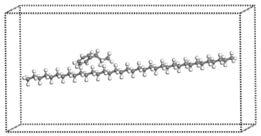<br>*d* = 3.87 Å<br>n-propylbenzene	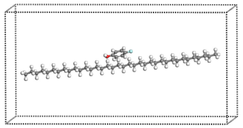<br>*d* = 3.41 Å<br>4-fluorophenol	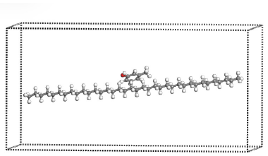<br>*d* = 3.33 Å<br>m-cresol	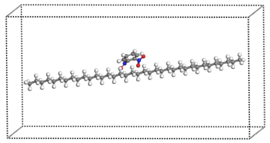<br>*d* = 3.57 Å<br>1,2-dinitrobenzene
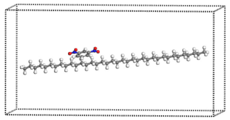<br>*d* = 3.28 Å<br>1,3-dinitrobenzene	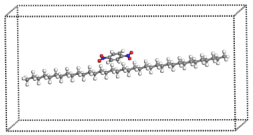<br>*d* = 3.48 Å<br>1,4-dinitrobenzene	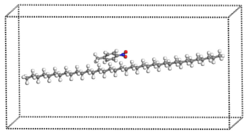<br>*d* = 3.49 Å<br>4-nitrotoluene	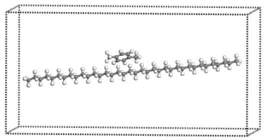<br>*d* = 3.88 Å<br>p-xylene	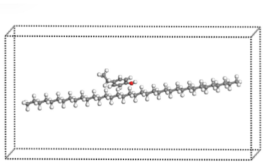<br>*d* = 3.62 Å<br>4-ethylphenol	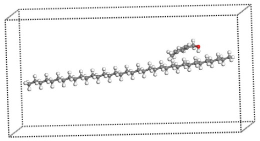<br>*d* = 3.72 Å<br>3-methylbenzyl alcohol
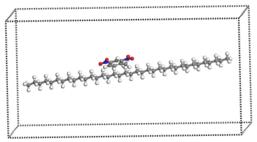<br>*d* = 3.33 Å<br>2,4-dinitrotoluene	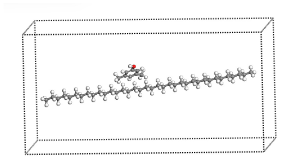<br>*d* = 3.48 Å<br>3,5-dimethylphenol	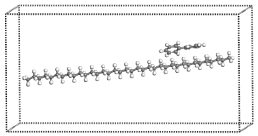<br>*d* = 3.50 Å<br>biphenyl	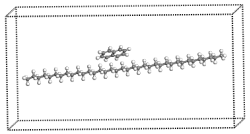<br>*d* = 3.96 Å<br>naphthalene	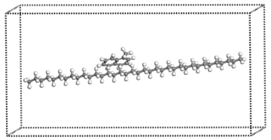<br>*d* = 3.52 Å<br>1-methylnaphthalene	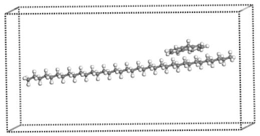<br>*d* = 3.46 Å<br>fluorene

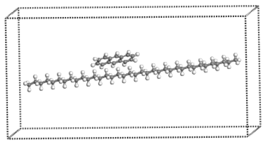<br>$d = 3.40$ Å<br>anthracene	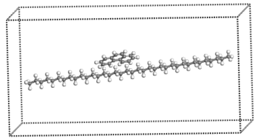<br>$d = 3.49$ Å<br>phenanthrene	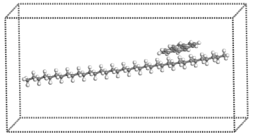<br>$d = 3.39$ Å<br>pyrene	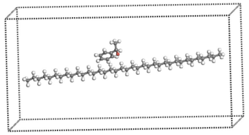<br>$d = 3.57$ Å<br>(1-bromoethyl)benzene	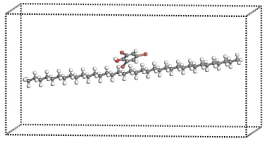<br>$d = 3.55$ Å<br>2,4,6-tribromophenol	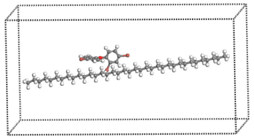<br>$d = 3.83$ Å<br>2,4,4'-tribromodiphenyl ether
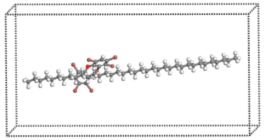<br>$d = 3.37$ Å<br>heptabromodiphenyl ether	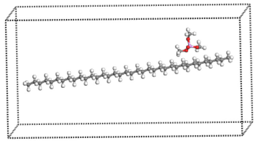<br>$d = 3.99$ Å<br>trimethyl phosphate	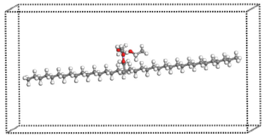<br>$d = 4.09$ Å<br>triethyl phosphate	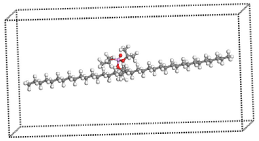<br>$d = 3.94$ Å<br>triisopropyl phosphate	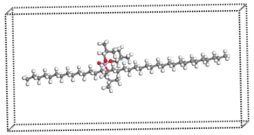<br>$d = 3.72$ Å<br>triisobutyl phosphate	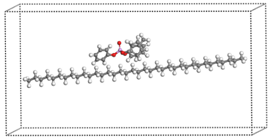<br>$d = 4.35$ Å<br>cresyl diphenyl phosphate
**POM**					
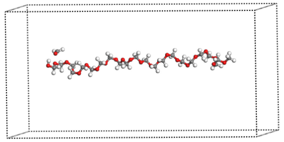<br>$d = 3.47$ Å<br>formaldehyde	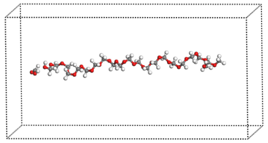<br>$d = 3.67$ Å<br>formic acid	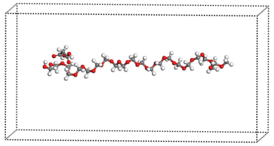<br>$d = 4.48$ Å<br>malonic acid	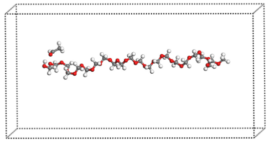<br>$d = 3.94$ Å<br>acetaldehyde	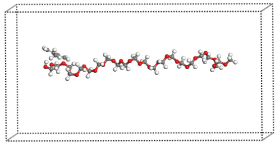<br>$d = 4.64$ Å<br>isoprene	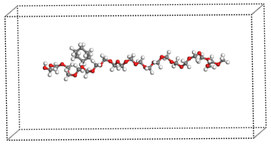<br>$d = 4.21$ Å<br>cyclohexane

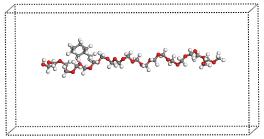<br>*d* = 4.21 Å<br>methylcyclohexane	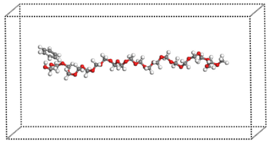<br>*d* = 3.72 Å<br>benzene	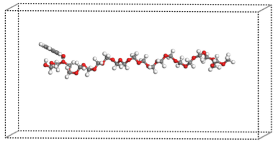<br>*d* = 3.76 Å<br>phenol	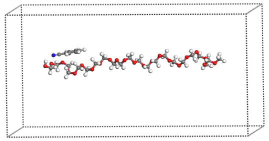<br>*d* = 3.71 Å<br>benzonitrile	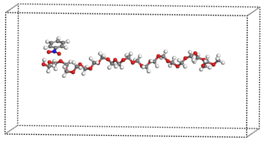<br>*d* = 3.65 Å<br>nitrobenzene	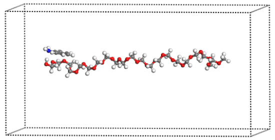<br>*d* = 3.94 Å<br>aniline
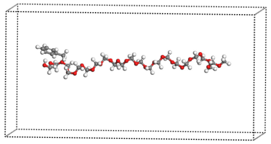<br>*d* = 3.65 Å<br>toluene	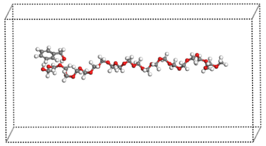<br>*d* = 3.73 Å<br>benzyl alcohol	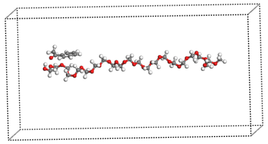<br>*d* = 4.26 Å<br>acetophenone	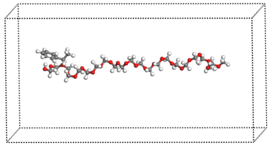<br>*d* = 3.76 Å<br>ethylbenzene	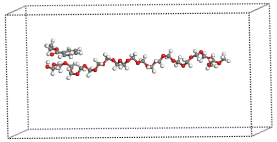<br>*d* = 4.22 Å<br>ethyl benzoate	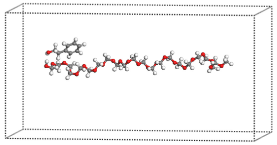<br>*d* = 4.75 Å<br>phenethyl alcohol
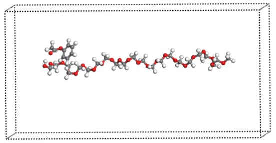<br>*d* = 4.78 Å<br>phenyl acetate	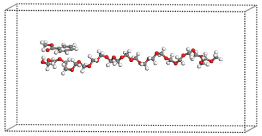<br>*d* = 4.24 Å<br>methyl benzoate	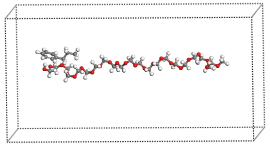<br>*d* = 3.98 Å<br>n-propylbenzene	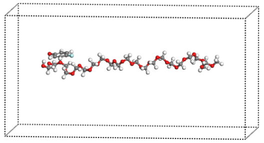<br>*d* = 4.37 Å<br>4-fluorophenol	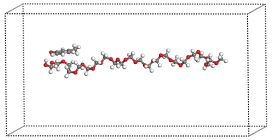<br>*d* = 4.36 Å<br>m-cresol	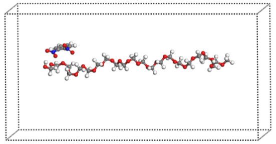<br>*d* = 3.95 Å<br>1,2-dinitrobenzene
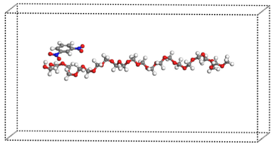<br>*d* = 3.80 Å<br>1,3-dinitrobenzene	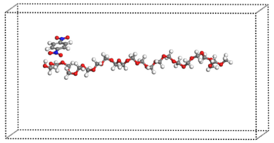<br>*d* = 3.89 Å<br>1,4-dinitrobenzene	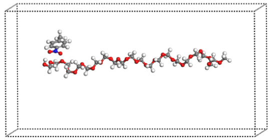<br>*d* = 3.70 Å<br>4-nitrotoluene	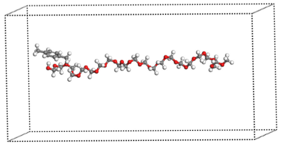<br>*d* = 3.79 Å<br>p-xylene	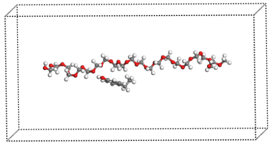<br>*d* = 3.47 Å<br>4-ethylphenol	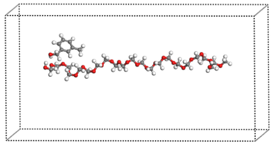<br>*d* = 4.27 Å<br>3-methylbenzyl alcohol

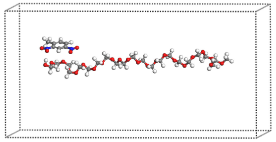<br>$d = 3.69$ Å<br>2,4-dinitrotoluene	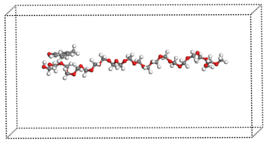<br>$d = 4.50$ Å<br>3,5-dimethylphenol	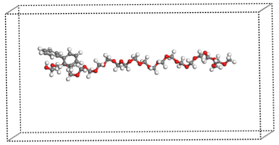<br>$d = 4.62$ Å<br>biphenyl	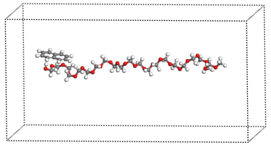<br>$d = 4.79$ Å<br>naphthalene	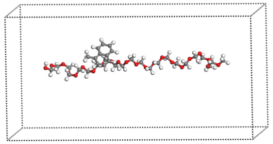<br>$d = 3.32$ Å<br>1-methylnaphthalene	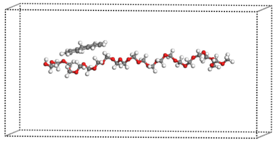<br>$d = 3.63$ Å<br>fluorene
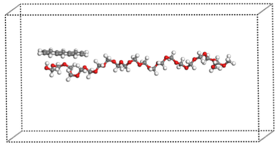<br>$d = 4.86$ Å<br>anthracene	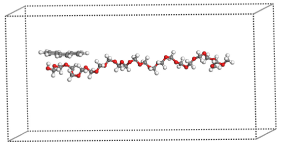<br>$d = 4.88$ Å<br>phenanthrene	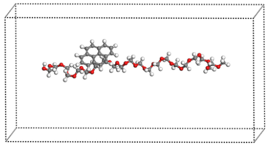<br>$d = 3.19$ Å<br>pyrene	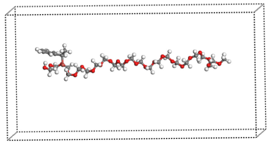<br>$d = 5.23$ Å<br>(1-bromoethyl)benzene	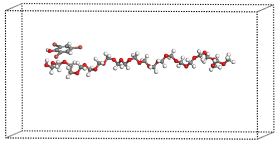<br>$d = 4.16$ Å<br>2,4,6-tribromophenol	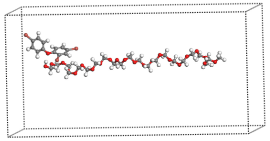<br>$d = 4.81$ Å<br>2,4,4'-tribromodiphenyl ether
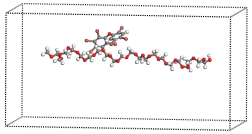<br>$d = 3.46$ Å<br>heptabromodiphenyl ether	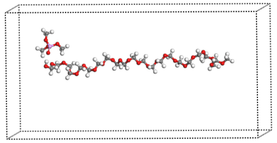<br>$d = 4.51$ Å<br>trimethyl phosphate	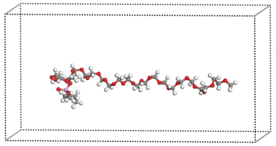<br>$d = 4.95$ Å<br>triethyl phosphate	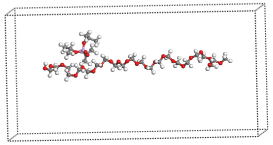<br>$d = 4.59$ Å<br>triisopropyl phosphate	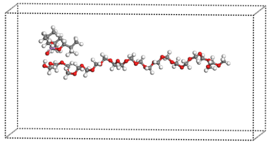<br>$d = 5.42$ Å<br>triisobutyl phosphate	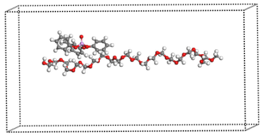<br>$d = 4.15$ Å<br>cresyl diphenyl phosphate
**PVA**					
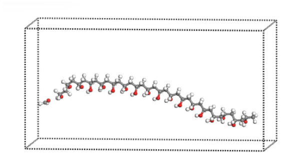<br>$d = 4.47$ Å<br>formaldehyde	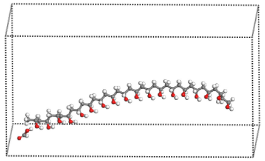<br>$d = 3.72$ Å<br>formic acid	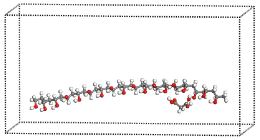<br>$d = 3.53$ Å<br>malonic acid	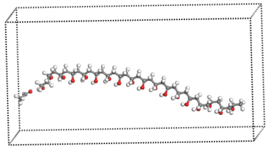<br>$d = 4.40$ Å<br>acetaldehyde	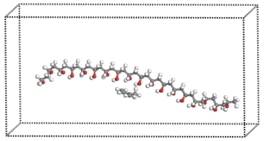<br>$d = 4.10$ Å<br>isoprene	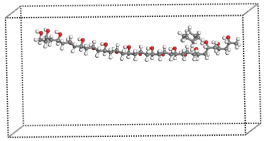<br>$d = 3.87$ Å<br>cyclohexane

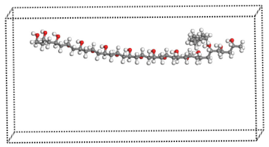<br>*d* = 4.11 Å<br>methylcyclohexane	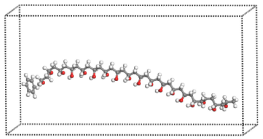<br>*d* = 3.88 Å<br>benzene	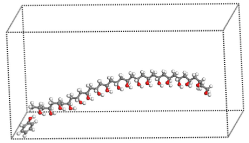<br>*d* = 4.51 Å<br>phenol	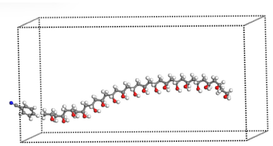<br>*d* = 5.41 Å<br>benzonitrile	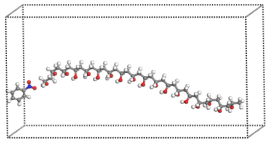<br>*d* = 5.43 Å<br>nitrobenzene	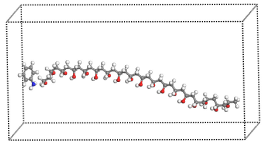<br>*d* = 3.63 Å<br>aniline
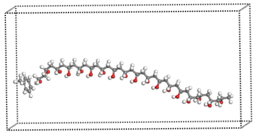<br>*d* = 3.70 Å<br>toluene	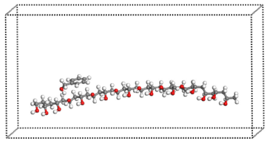<br>*d* = 3.50 Å<br>benzyl alcohol	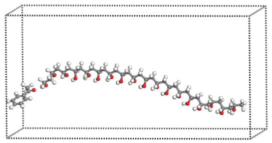<br>*d* = 5.49 Å<br>acetophenone	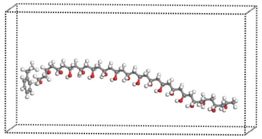<br>*d* = 3.67 Å<br>ethylbenzene	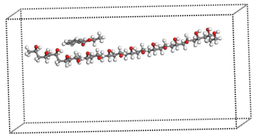<br>*d* = 3.71 Å<br>ethyl benzoate	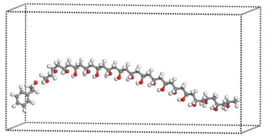<br>*d* = 5.21 Å<br>phenethyl alcohol
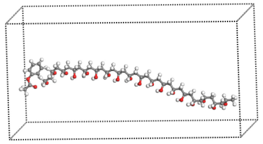<br>*d* = 3.61 Å<br>phenyl acetate	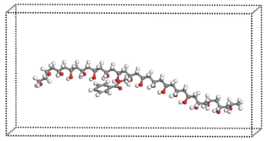<br>*d* = 4.17 Å<br>methyl benzoate	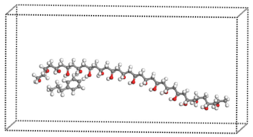<br>*d* = 4.24 Å<br>n-propylbenzene	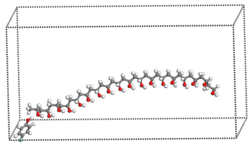<br>*d* = 4.89 Å<br>4-fluorophenol	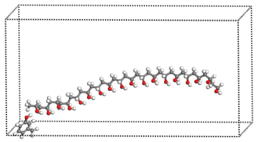<br>*d* = 5.03 Å<br>m-cresol	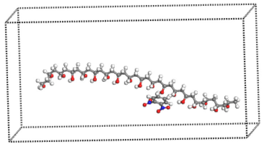<br>*d* = 4.28 Å<br>1,2-dinitrobenzene
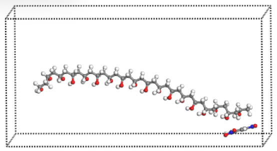<br>*d* = 3.94 Å<br>1,3-dinitrobenzene	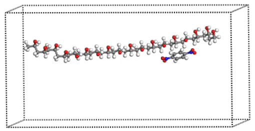<br>*d* = 3.41 Å<br>1,4-dinitrobenzene	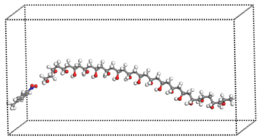<br>*d* = 5.91 Å<br>4-nitrotoluene	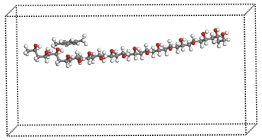<br>*d* = 3.95 Å<br>p-xylene	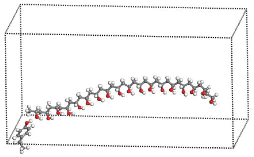<br>*d* = 5.23 Å<br>4-ethylphenol	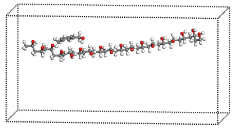<br>*d* = 3.49 Å<br>3-methylbenzyl alcohol

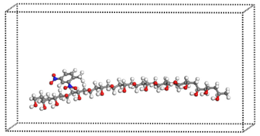<br>*d* = 3.66 Å<br>2,4-dinitrotoluene	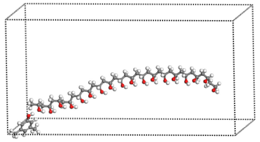<br>*d* = 4.85 Å<br>3,5-dimethylphenol	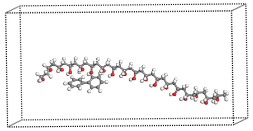<br>*d* = 4.33 Å<br>biphenyl	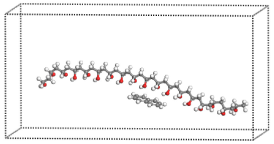<br>*d* = 4.42 Å<br>naphthalene	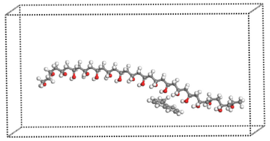<br>*d* = 4.48 Å<br>1-methylnaphthalene	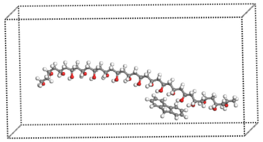<br>*d* = 4.32 Å<br>fluorene
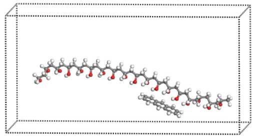<br>*d* = 4.35 Å<br>anthracene	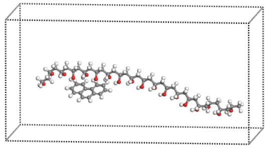<br>*d* = 4.34 Å<br>phenanthrene	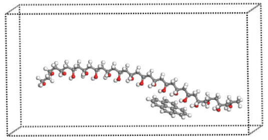<br>*d* = 4.30 Å<br>pyrene	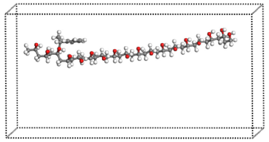<br>*d* = 3.81 Å<br>(1-bromoethyl)benzene	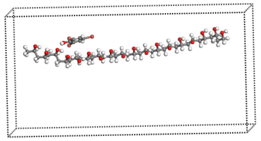<br>*d* = 3.69 Å<br>2,4,6-tribromophenol	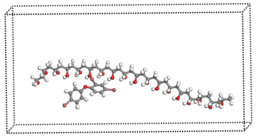<br>*d* = 4.89 Å<br>2,4,4'-tribromodiphenyl ether
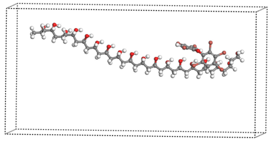<br>*d* = 4.18 Å<br>heptabromodiphenyl ether	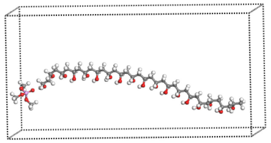<br>*d* = 5.03 Å<br>trimethyl phosphate	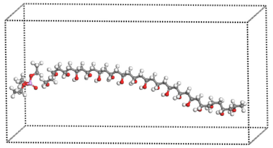<br>*d* = 4.65 Å<br>triethyl phosphate	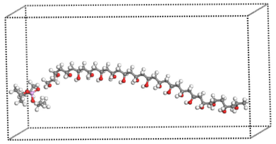<br>*d* = 5.01 Å<br>triisopropyl phosphate	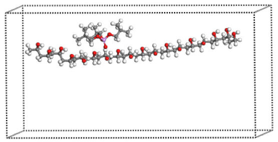<br>*d* = 4.59 Å<br>triisobutyl phosphate	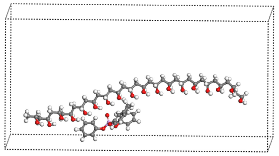<br>*d* = 5.16 Å<br>cresyl diphenyl phosphate

d represents the distance between the center of molecule and the nearest carbon atom in PE, POM or PVA nanoplastics.

Table S4. Standardized coefficients, *t*, *p* values and variable inflation factor (*VIF*) for the predictive variables

QSAR models for log*K* values												
	PE				POM				PVA			
Descriptors	Standardized coefficients	*t**	*p**	*VIF*	Standardized coefficients	*t**	*p**	*VIF*	Standardized coefficients	*t**	*p**	*VIF*
ATSC0p	0.800	14.783	< 0.001	2.365	0.807	12.045	< 0.001	2.365	0.817	11.314	< 0.001	2.365
SpMax1_Bhm	0.349	6.935	< 0.001	2.048	0.339	5.441	< 0.001	2.048	0.346	5.152	< 0.001	2.048
LipoaffinityIndex	–0.248	–6.079	< 0.001	1.349	–0.284	–5.606	< 0.001	1.349	–0.384	–7.039	< 0.001	1.349
QSAR models for C_{m} values												
	PA				PC				PE			
Descriptors	Standardized coefficients	*t**	*p**	*VIF*	Standardized coefficients	*t**	*p**	*VIF*	Standardized coefficients	*t**	*p**	*VIF*
AATS5m	–0.407	–4.972	< 0.001	5.26	–0.410	–5.019	< 0.001	5.26	–0.117	–0.758	< 1	5.26
MATS8c	0.017	0.400	< 1	1.381	0.002	0.053	< 1	1.381	0.895	11.347	< 0.001	1.381
GATS8c	0.029	0.676	< 1	1.456	0.036	0.837	< 1	1.456	–0.469	–5.786	< 0.001	1.456
VE1_DzZ	–0.074	–2.034	< 0.1	1.037	–0.071	–1.957	< 0.1	1.037	–0.207	–3.031	< 0.01	1.037
AMW	1.311	16.186	< 0.001	5.150	1.312	16.236	< 0.001	5.150	0.67	4.399	< 0.001	5.150
	PP				PS				PU			
AATS5m	–0.378	–3.291	< 0.01	5.26	–0.407	–2.523	< 0.1	5.26	–0.413	–5.073	< 0.001	5.26
MATS8c	0.021	0.353	< 1	1.381	0.017	0.208	< 1	1.381	0.006	0.154	< 1	1.381
GATS8c	0.035	0.579	< 1	1.456	0.036	0.424	< 1	1.456	0.024	0.558	< 1	1.456
VE1_DzZ	–0.115	–2.247	< 0.1	1.037	–0.047	–0.661	< 1	1.037	–0.07	–1.926	< 0.1	1.037
AMW	1.252	11.019	< 0.001	5.150	1.229	7.694	< 0.001	5.150	1.316	16.33	< 0.001	5.150
	PAA				PET				POM			
AATS5m	–0.407	–5.348	< 0.001	5.26	–0.517	–6.416	< 0.001	5.26	–0.328	–3.666	< 0.01	5.26
MATS8c	0.006	0.155	< 1	1.381	0.012	0.286	< 1	1.381	0.009	0.205	< 1	1.381

GATS8c	0.021	0.518	< 1	1.456	0.027	0.642	< 1	1.456	0.026	0.556	< 1	1.456
VE1_DzZ	–0.066	–1.956	< 0.1	1.037	–0.078	–2.167	< 0.1	1.037	–0.088	–2.202	< 0.1	1.037
AMW	1.316	17.458	< 0.001	5.150	1.399	17.533	< 0.001	5.150	1.238	13.97	< 0.001	5.150
	PVC				PVA				PMMA			
AATS5m	–0.385	–4.964	< 0.001	5.26	–0.375	–2.761	< 0.01	5.26	–0.459	–5.902	< 0.001	5.26
MATS8c	0.005	0.130	< 1	1.381	0.027	0.390	< 1	1.381	0.014	0.361	< 1	1.381
GATS8c	0.033	0.803	< 1	1.456	0.039	0.539	< 1	1.456	0.031	0.754	< 1	1.456
VE1_DzZ	–0.081	–2.340	< 0.1	1.037	–0.141	–2.33	< 0.1	1.037	–0.08	–2.322	< 0.1	1.037
AMW	1.293	16.842	< 0.001	5.150	1.222	9.09	< 0.001	5.150	1.354	17.609	< 0.001	5.150

Table S5. Definitions for the descriptors utilized in the developed models

Descriptors	Definitions (Yap, 2011)
ATSC0p	Centered Broto-Moreau autocorrelation - lag0/weighted by polarizabilities
SpMax1_Bhm	Largest absolute eigenvalue of Burden modified matrix - n1/weighted by relative mass
LipoaffinityIndex	Lipoaffinity index
AATS5m	Average Broto-Moreau autocorrelation - lag5/weighted by mass
MATS8c	Moran autocorrelation - lag8/weighted by charges
GATS8c	Geary autocorrelation - lag 8/weighted by charges
VE1_DzZ	Coefficient sum of the last eigenvector from Barysz matrix/weighted by atomic number
AMW	Average molecular weight (Molecular weight/Total number of atoms)
GATS3v	Geary autocorrelation - lag3/weighted by van der Waals volumes
SIC2	Structural information content index (neighborhood symmetry of 2-order)
BIC3	Bond information content index (neighborhood symmetry of 3-order)

Table S6. Hammett parameters of substituents for 10 different organic compounds and their adsorption energies (E_{ad}) on PE, POM and PVA nanoplastics

No.	Compound	substituents	σ_m	σ_p	E_{ad} (kcal/mol)		
					PE	POM	PVA
1	cyclohexane				–6.37	–8.18	–8.52
2	benzene				–7.36	–8.54	–7.44
3	benzyl alcohol	$-CH_2OH$	0	0	–9.79	–14.81	–16.11
4	toluene	$-CH_3$	–0.07	–0.17	–9.36	–10.69	–8.12
5	ethylbenzene	$-CH_2CH_3$	–0.07	–0.15	–9.14	–11.15	–10.24
6	n-propylbenzene	$-CH_2CH_2CH_3$	–0.07	–0.13	–9.04	–11.67	–11.15
7	aniline	$-NH_2$	–0.16	–0.66	–10.06	–13.80	–11.65
8	phenol	-OH	0.12	–0.37	–8.67	–15.62	–12.83
9	benzonitrile	-CN	0.56	0.66	–7.65	–13.24	–11.90
10	nitrobenzene	$-NO_2$	0.71	0.78	–8.86	–8.20	–10.41

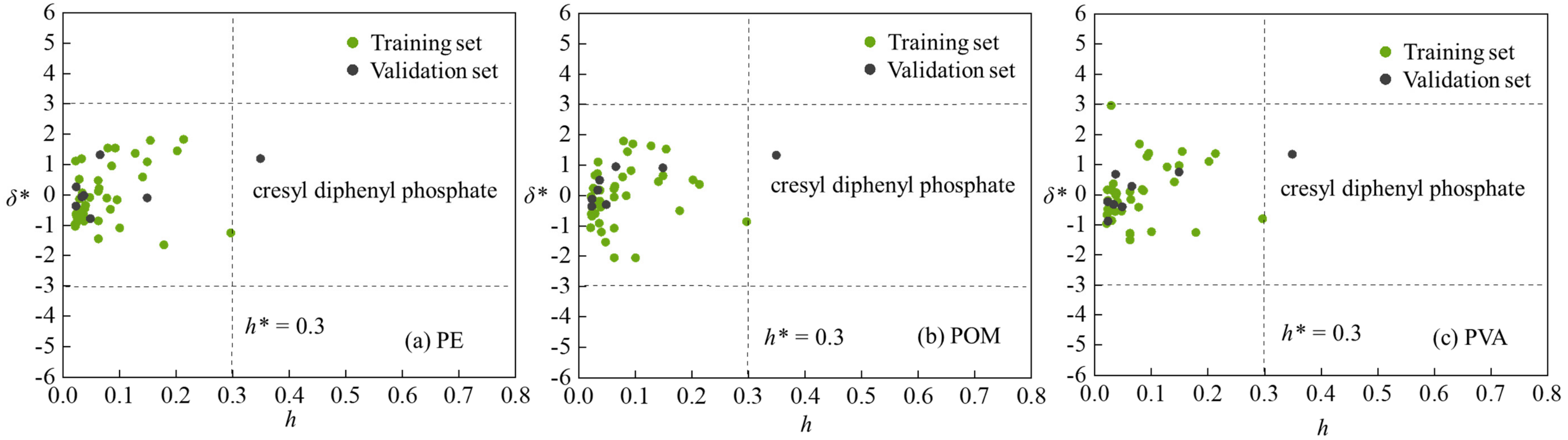

Figure S1. Application domain characterized by Williams plots with standardized residuals (δ*) and leverage values (*h*) for QSAR models of log*K* values on (a) PE, (b) POM and (c) PVA nanoplastics (*h**: the warning leverage value)

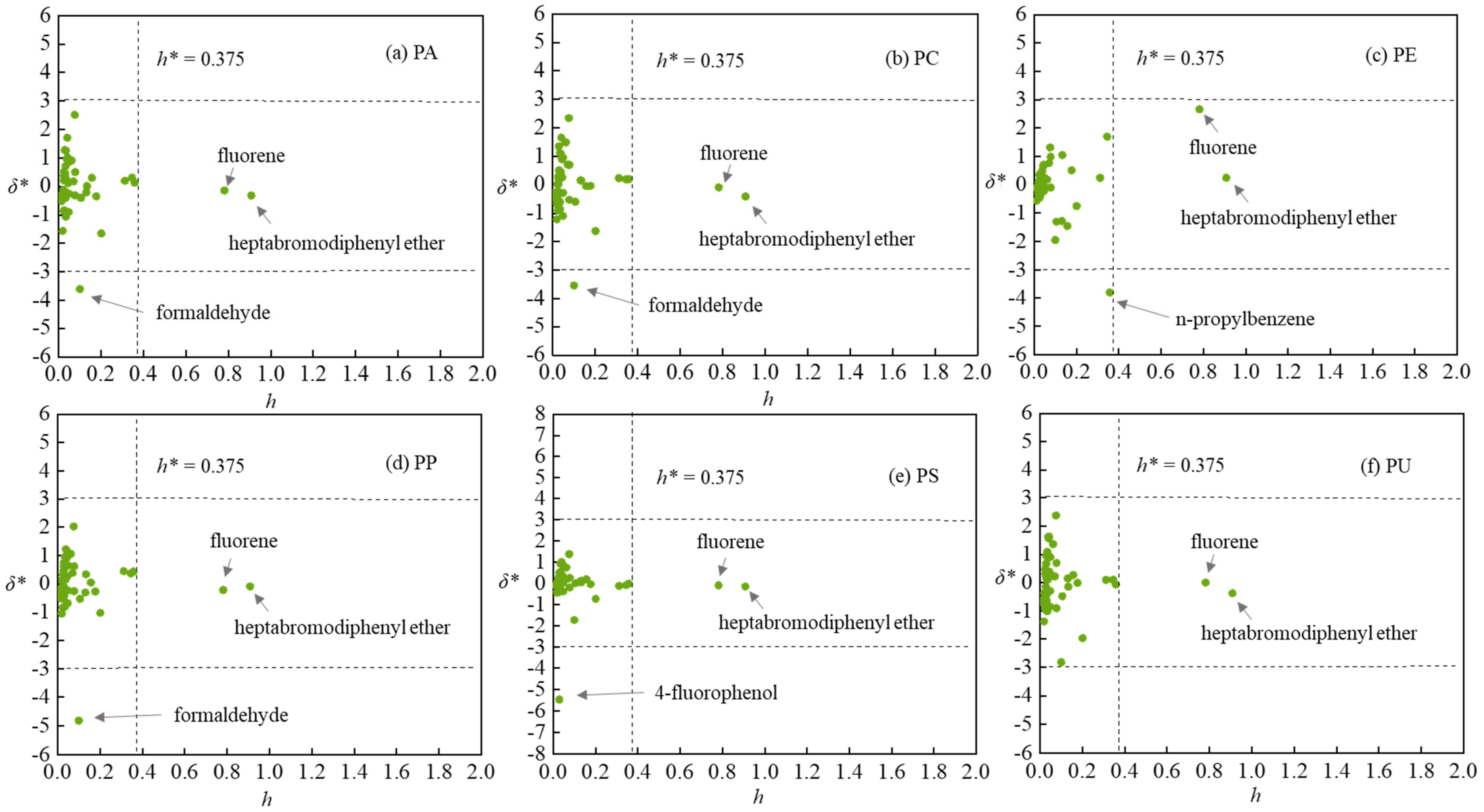

Figure S2. Application domain characterized by Williams plots with standardized residuals (δ^*) and leverage values (h) for QSAR models of C_m values on (a) PA, (b) PC, (c) PE, (d) PP, (e) PS and (f) PU nanoplastics (h^*: the warning leverage value)

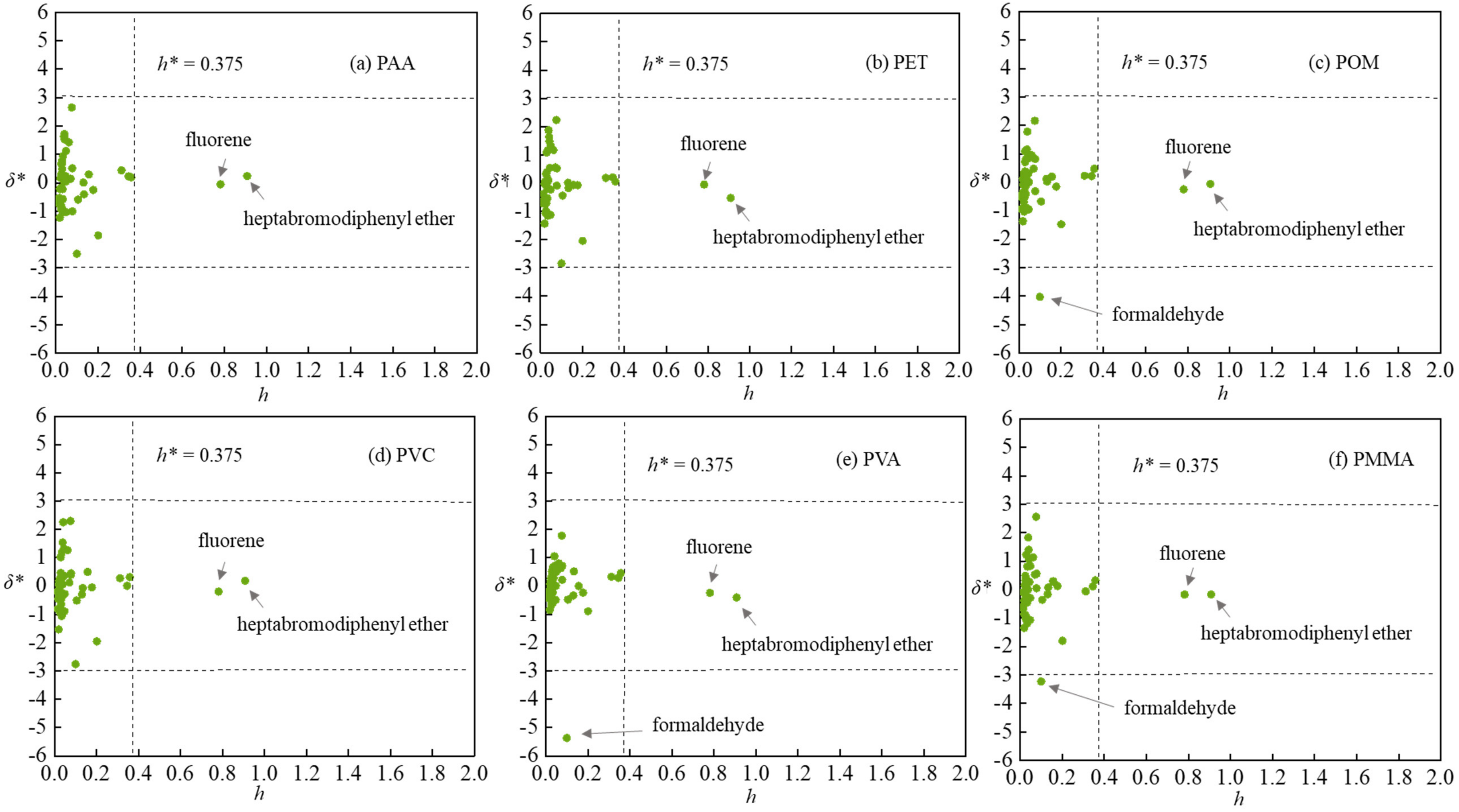

Figure S3. Application domain characterized by Williams plots with standardized residuals (δ^*) and leverage values (h) for QSAR models of C_m values on (a) PAA, (b) PET, (c) POM, (d) PVC, (e) PVA and (f) PMMA nanoplastics (h^*: the warning leverage value)

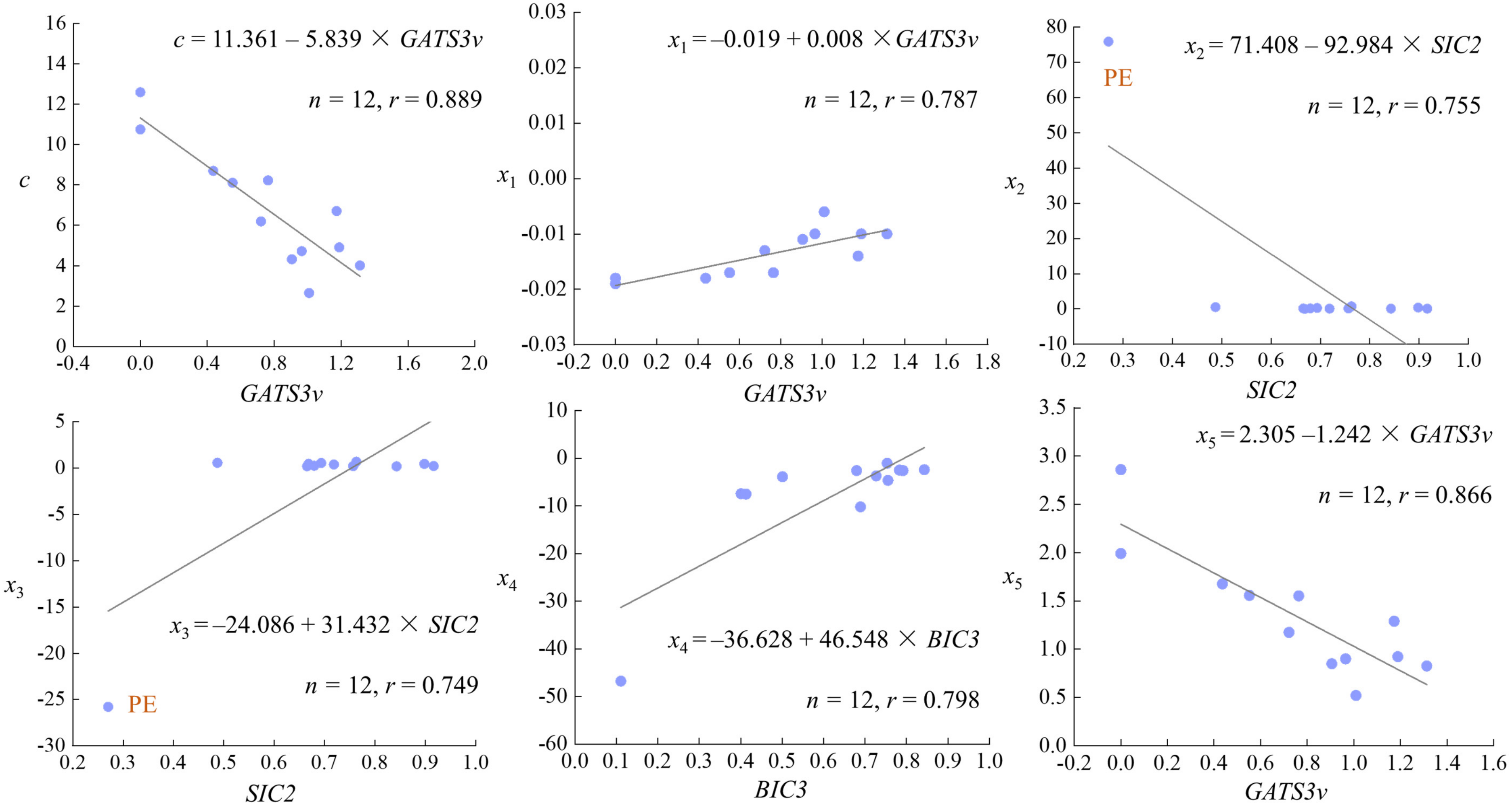

Figure S4. Prediction for c, x_1, x_2, x_3, x_4 and x_5 based on descriptors characterizing nanoplastics (r is the correlation coefficient)
